# scCirclehunter delineates ecDNA-containing cells using single-cell ATAC-seq, with a focus on glioblastoma

**DOI:** 10.1038/s41421-025-00842-9

**Published:** 2025-12-09

**Authors:** Rong Jiang, Zhengmao Lu, Fang Li, Yibei Zhu, Manqiu Yang, Shufan Zhang, Ping Wu, Chengliang Gong, Yiyuan Fei, Yonghua Sang, Yulun Huang, Jiong Jiong Guo, Moli Huang

**Affiliations:** 1 MOE Key Laboratory of Geriatric Diseases and Immunology, School of Basic Medical Sciences, Soochow University, Suzhou, Jiangsu China; 2 Department of Gastrointestinal Surgery, Shanghai Changhai Hospital, Naval Medical University, Shanghai, China; 3 School of Biology Sciences, Soochow University, Suzhou, Jiangsu China; 4 Department of Cardiothoracic Surgery, The Second Affiliated Hospital of Soochow University, Suzhou, Jiangsu China; 5 Department of Neurosurgery, Dushu Lake Hospital Affiliated of Soochow University, Suzhou, Jiangsu China; 6 Department of Orthopedics and Sports Medicine, The First Affiliated Hospital of Soochow University, Suzhou, Jiangsu China

**Keywords:** Tumour heterogeneity, Oncogenes, Cancer genomics, Bioinformatics, Epigenetics analysis

## Abstract

In cancer, extrachromosomal DNA (ecDNA) contributes to tumor heterogeneity and is associated with poor prognosis, but studies on patient-derived ecDNA are relatively limited at single-cell resolution. Here, we introduce scCirclehunter, a framework designed to identify ecDNA from scATAC-seq data and assign ecDNA to specific cell populations. Leveraging scCirclehunter and available glioblastoma (GBM) datasets, we uncover the inter-cellular heterogeneity of ecDNA-carrying cells across GBM patients and trace the trajectories of malignant cells within a single patient that harbors multiple ecDNAs. By integrating scRNA-seq data, we use ecNR2E1 as an example to demonstrate that ecDNA drives tumor progression in GBM through several mechanisms. Additionally, our findings suggest a potential link between ecDNA and increased mitochondrial transfer frequency. Overall, scCirclehunter provides a novel framework for analyzing patient-specific ecDNAs with single-cell precision, offering insights into the role of ecDNA-carrying cells in driving GBM heterogeneity.

## Introduction

Extrachromosomal DNA (ecDNA) refers to DNA circles that are separate from linear chromosomes and typically range in size from several hundred kilobases to a few megabases. These ecDNAs frequently harbor oncogenes along with regulatory elements, promoting elevated oncogene expression^1^. Current research on ecDNA has largely focused on its commonalities, such as its association with tumorigenesis, drug resistance, and tumor subtypes^2–4^. Investigations into the regulatory mechanisms of ecDNA have primarily relied on well-established cell lines, such as COLO320DM, GBM39, PC3, and others^5–7^. Although substantial progress has been made in elucidating the role of ecDNA using cell lines, such studies often fail to capture the complex regulatory dynamics and transcriptional heterogeneity observed in patient-derived samples. A notable example is the variation in copy number of ecDNA carrying *MYC* family genes, which has been shown to drive transcriptional heterogeneity among different cells within patients with neuroblastoma, small-cell lung cancer, and pancreatic ductal adenocarcinoma^2,8,9^. In neuroblastoma, for instance, cells with high copies of ecMYCN exhibited increased expression of MYCN target genes, activated ribosome biogenesis pathway, and downregulated cell‒cell interaction pathway, compared to cells with lower copies of ecMYCN^8^. Such studies, particularly at single-cell resolution, are currently lacking, leaving a critical gap in understanding how ecDNA shapes intratumoral heterogeneity within patients.

The unique circular structure and active transcription of ecDNAs result in higher chromatin accessibility compared to linear chromosomes, enabling the identification of ecDNA using an assay for transposase-accessible chromatin with sequencing (ATAC-seq)^7^. In our previous research, we developed circlehunter, a tool designed to identify ecDNA based on bulk ATAC-seq data^10^. However, bulk sequencing signals typically originate from thousands of heterogeneous cells within patients, which means that the observed signals may derive from both ecDNA-carrying cancer cells and cells lacking ecDNA^1^. Additionally, individual patients often lack controls, and interpatient variability is influenced by many factors besides ecDNA. These limitations complicate the interpretation of ecDNA’s contribution to tumor heterogeneity and constrain the utility of ATAC-seq in ecDNA studies. In recent years, the application of single-cell technologies to investigate heterogeneity has greatly advanced our understanding of various aspects of cancer. In particular, single-cell ATAC-seq (scATAC-seq) has proven effective in studying cell-type specific regulatory mechanisms^11^. By establishing regulatory relationships between accessible genomic elements and genes, scATAC-seq offers a powerful tool for examining transcriptional and regulatory differences between ecDNA-carrying cells and those without ecDNA. This approach provides new opportunities to elucidate the role of ecDNA in shaping tumor heterogeneity at the single-cell level.

Glioblastoma (GBM) is among the deadliest cancers, with ecDNA occurrence observed in ~60% of cases^12^. Isocitrate dehydrogenase wild type (IDH-wt) GBM is the most prevalent form of the disease^13^. The tumor and immune microenvironment heterogeneity in GBM is a major cause of treatment resistance, with ecDNA playing a key role in promoting tumor heterogeneity^2^. Genes such as *EGFR*, *PDGFRA*, *CDK4*, *MDM2*, and *MDM4* are frequently amplified in GBM patients and are commonly located on ecDNAs^12,14^. Given the widespread prevalence of ecDNA and its association with tumor heterogeneity, exploring its role in GBM is essential for understanding how ecDNA amplifications drive both intratumoral and intertumoral heterogeneity, and for advancing the development of ecDNA-targeted therapies.

Here, we present the scCirclehunter, a novel framework tailored for scATAC-seq data, which not only identifies ecDNA with a pseudo-bulk strategy but assigns candidate ecDNA to specific cell populations. This approach enables the investigation of ecDNA-driven heterogeneity at a single-cell level, offering unique insights into their regulatory roles. In this study, we leverage scCirclehunter to investigate ecDNA heterogeneity across different GBM patients and within distinct spatial regions of individual tumors. Moreover, we explore the relationships between multiple ecDNAs within a GBM patient. By integrating paired scATAC-seq and scRNA-seq data, we elucidate how ecDNA aberrantly activates regulatory networks, contributing to tumor heterogeneity and therapy resistance. Lastly, we propose that ecDNA may be associated with an increased frequency of mitochondrial transfer.

## Results

### scCirclehunter: assigning ecDNA to cell populations

Recent studies have shown that the amplification of ecDNA harboring oncogene significantly contributes to tumor heterogeneity^2^. However, research on the regulatory mechanisms of ecDNA at the single-cell level remains limited. Moreover, most scATAC-seq-based copy number inference methods typically operate on fixed-step bins without considering the boundaries of ecDNA, which restricts the accurate study of cells carrying ecDNA^15^. To address this gap, building upon our previously developed method for identifying ecDNA in ATAC-seq libraries^10^, we introduce a framework named scCirclehunter. This approach enables the detection of ecDNA from scATAC-seq data and assigns ecDNA to cell populations, allowing for a detailed study of the heterogeneity and regulatory mechanisms of ecDNA-containing cells. The assumptions underlying this method are as follows: (1) compared to linear amplification or non-amplified mechanisms, ecDNA typically exhibits more widespread copies; (2) ecDNA regions mapped to the genome display continuous chromatin accessibility signals; (3) the selective advantage conferred by ecDNA makes it the primary contributor to gene copies in patients where ecDNA is present. Previous studies based on scDNA-seq and scATAC-seq have independently validated that ecDNA exhibits more extensive copies and chromatin accessibility signals than linear amplification^2,16^.

**Fig. 1:**
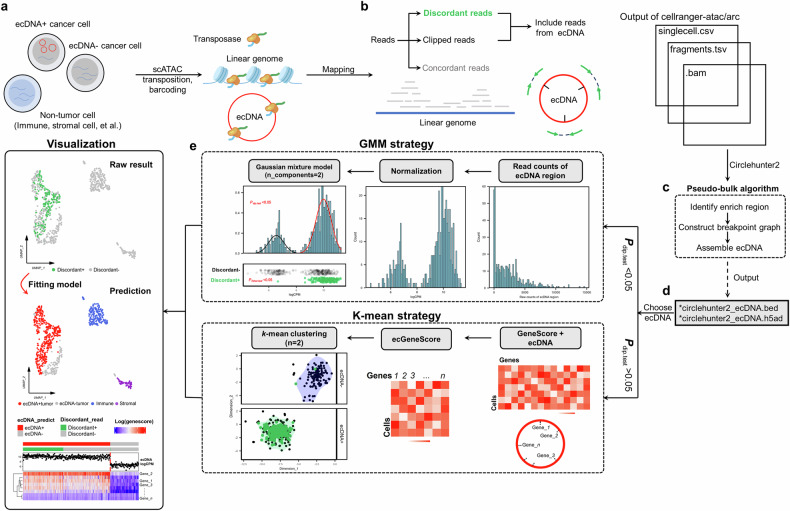
scCirclehunter framework. **a** Tumor tissues are dissociated, and scATAC-seq libraries are constructed. **b** Reads derived from either ecDNA or linear chromosomes are then mapped to the genome. **c** A pseudo-bulk algorithm is applied to identify ecDNA regions within the scATAC-seq library. **d** The normalized ATAC-seq signals are assessed to determine whether they follow a bimodal mixture model. When *P* value < 0.05, it indicates that there are multiple normal distributions. **e** ecDNA is assigned to specific cell populations using the GMM or *k*-mean clustering.

Using the 10X scATAC-seq library as an example, the scCirclehunter framework can be summarized as follows. Tumor tissues, consisting of malignant cells (with or without ecDNA) and other cells, are dissociated into single cells and captured in droplets. Afterward, the transposase inserts sequencing adapters into the open chromatin regions of both chromosomal DNA and ecDNA circles (Fig. 1a). Although sequencing depth for individual cells is limited, the scATAC-seq library typically includes hundreds of qualified cells. Given the amplification of ecDNA, there is a high likelihood that the library can capture sufficient reads from the accessible chromatin regions of ecDNA, which are not wrapped in nucleosomes. Next, all reads are mapped to the genome, and discordant or clipped reads originating from the ecDNA breakpoints provide valuable insights into the ecDNA boundaries and the connections between ecDNA segments (Fig. 1b). A pseudo-bulk algorithm is then applied to identify candidate ecDNAs in the scATAC-seq library (Fig. 1c)^10^. Subsequently, a multimodal test is performed on the normalized ATAC signal within the selected ecDNA regions across all cells (Fig. 1d). Cells containing discordant reads at the ecDNA breakpoints that indicate circular structures are then extracted. These cells with discordant reads serve as the gold standard for classifying cells carrying ecDNAs. Finally, we fit the normalized ATAC signals within the ecDNA region across all cells using a Gaussian mixture model (GMM), a method widely used to classify cells with amplification^15,17,18^. Using the gold standard cells as a reference, we perform a Fisher’s exact test to determine whether cells from the second distribution with a higher mean are more likely to carry discordant reads (Fig. 1e). A detailed description of the method is provided in the “Methods” section and Supplementary Fig. S1.

### Performance of scCirclehunter

**Fig. 2:**
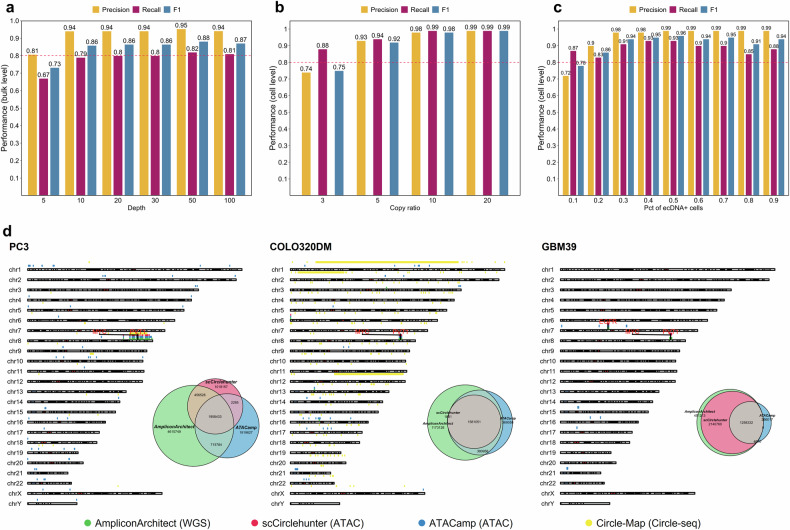
scCirclehunter performance based on simulated and real data. **a** Precision, recall, and F1 score of ecDNA detection with varying mock ecDNA local depth at 50 bp read length. **b** Precision, recall, and F1 score of GMM in discriminating cells with simulated local amplification, across varying amplification copy ratios. **c** Precision, recall, and F1 score of scCirclehunter in distinguishing ecDNA^+^ SW480 cells from ecDNA^‒^ DLD1 cells when setting different proportions of ecDNA^+^ cells. **d** Karyotype plots depicting genomic distributions of circular regions detected across all tools. Euler diagrams visualizing overlap among regions identified by AA, scCirclehunter, and ATACamp.

To evaluate the performance of our framework, we conducted two sets of tests: (1) assessing the capability to identify ecDNA from scATAC-seq libraries, and (2) assessing the models’ ability to distinguish cells carrying ecDNA focal amplifications. In the initial test, we simulated 100 ecDNAs and used the 10X Genomics PBMC1k dataset, a benchmark scATAC-seq resource comprising 1004 qualified nuclei from a healthy donor’s peripheral blood mononuclear cells (PBMCs), as background chromatin accessibility signals (mean sequencing depth = 4×). When the simulated ecDNA depth was set to 5×, slightly higher than the average depth of PBMC1k, the precision was approximately 0.8. However, as the ecDNA depth increased to ≥ 10×, significantly surpassing the genome-wide average, the method consistently achieved a precision of 0.94 and an F1-score of 0.86 (Fig. 2a), highlighting its robustness in identifying ecDNA using a pseudo-bulk strategy. Building on this, we further focused on the ability of GMM to distinguish cells with ecDNA focal amplifications. To this end, we simulated the bin-by-cell count matrix for cells with ecDNA focal amplifications using simATAC^15,19^, adjusting the local amplification depth by multiplying the copy ratio with the Poisson distribution parameter. Simulated ecDNAs ranged in size from 500 kb to 10 Mb. Overall, the GMM consistently demonstrated high recall, achieving a value of 0.9. However, for ecDNA with low copy numbers (copy ratio = 3), precision decreased, indicating the difficulty of distinguishing cells with ecDNA from those without at low depths. At higher depths (copy ratio ≥ 5), the GMM achieved an F1-score exceeding 0.9 (Fig. 2b). A critical challenge in identifying focal amplifications from scATAC-seq data lies in accurately segmenting the genome and precisely identifying amplified regions^15,17^. Accurately extracting ecDNA regions can reduce noise in classifying cells with ecDNA focal amplifications, as ecDNA regions typically show significantly increased copy numbers and accessibility signals.

To further evaluate the model’s capability using real data, we mixed a cell line containing ecDNA with a corresponding ecDNA-negative cell line of the same tumor type. We then assessed the model’s ability to distinguish ecDNA^+^ from ecDNA^‒^ cells based on the ecDNA region identified in the upstream analysis. Here, we selected two colon adenocarcinoma cell lines: SW480 (ecDNA^+^) and DLD1 (ecDNA^‒^). WGS data revealed that SW480 carried ecMYC, whereas DLD1 did not harbor ecDNA (Supplementary Fig. S2a). Based on 10X scATAC-seq data, we identified a 3.46 Mb ecDNA containing *MYC* and *PVT1*, consistent with the high-copy seed intervals identified by AA (Supplementary Fig. S2b‒c). When analyzing data that pooled several cell lines for each scATAC-seq run from Zhu et al., ecMYC was successfully detected in reads derived from SW480 cells, while *PVT1* was not observed (Supplementary Fig. S2d). We speculated that this discrepancy may be due to insufficient depth in the SW480 cells caused by the mixed sequencing of multiple cell lines, or due to the presence of alternative ecDNA structures. Notably, a similar ecMYC structure lacking *PVT1* was also identified in the 10X SW480 library (Supplementary Fig. S2e). To further assess the model’s robustness, we mixed 1000 SW480 and DLD1 cells, gradually increasing the proportion of ecDNA^+^ SW480 cells from 10% to 90%. Based on the identified ecMYC region, our model achieved an average precision of 0.94, recall of 0.89, and F1 score of 0.91 in distinguishing SW480 (ecMYC^+^) from DLD1 (ecMYC^‒^) cells, demonstrating the strategy’s reliability in real-world applications (Fig. 2c).

The amplification of ecDNA is more heterogeneous in DNA copy number among cells than homogeneously staining regions (HSRs), as evidenced by copy number estimates from scATAC-seq data in COLO320DM (median CN = 24, maximum CN = 237) and COLO320HSR cells (median CN = 18, maximum CN = 58) (Supplementary Fig. S2f). Consequently, the GMM may be capable of distinguishing COLO320DM from COLO320HSR cells, given the known ecMYC regions. To test this, we fitted two distributions to the normalized signal of the ecMYC region in a mixed population of COLO320DM and COLO320HSR cells. The means of the fitted distributions closely aligned with the true values (true mean for COLO320HSR = 9.91, COLO320DM = 10.31; predicted mean for COLO320HSR = 9.91, COLO320DM = 10.28), and the predicted composition was also consistent with the true proportions (true COLO320HSR/DM = 0.597/0.403, predicted COLO320HSR/DM = 0.556/0.444). Overall, the GMM achieved a precision of 0.75 (Supplementary Fig. S2g). A previous study has reported that not all cells within a cell line exhibit uniform ecDNA amplification, highlighting ecDNA heterogeneity within the cell line^20^. The heterogeneity may partially account for the lack of a well-defined multimodal distribution in the ecMYC region for the COLO320HSR and COLO320DM cells. To overcome this limitation, we applied k-means clustering using the ecDNA Gene Score as an alternative approach. This method achieved a precision of 0.84 and an F1-score of 0.80 in distinguishing COLO320DM from COLO320HSR cells, without requiring assumptions about the underlying data distribution (Supplementary Fig. S2h, i).

We also compared scCirclehunter with other tools, including Circle-Map^21^, AA, and ATACamp^22^. Circle-Map was applied to Circle-seq data, AA to WGS data, and scCirclehunter and ATACamp to bulk ATAC-seq data. Results from PC3 and COLO320DM cell lines revealed that AA, scCirclehunter, and ATACamp exhibited concordance in detecting high-copy ecDNA regions. In contrast, Circle-Map primarily identified small, single-fragment eccDNAs (detecting 5049 and 31,594 eccDNAs in PC3 and COLO320DM, respectively, before filtering). In PC3, AA, scCirclehunter, and ATACamp uniformly detected *MYC* amplification. For COLO320DM, all three tools consistently identified MYC-containing ecDNA fragments and fragments from chromosome 6. However, scCirclehunter failed to detect short fragments on chromosomes 13 and 16. In GBM39, both scCirclehunter and AA detected ecMYC and ecEGFR amplifications, whereas ATACamp captured ecEGFR but missed ecMYC. Collectively, these tools primarily target amplified ecDNA regions and can effectively identify core ecDNA amplifications (Fig. 2d). Furthermore, scCirclehunter achieved relatively precise ecDNA breakpoint estimates compared to ATACamp. Using AA-inferred breakpoints as the reference standard, we annotated breakpoints in ecDNA fragments carrying key oncogenes within GBM39, PC3, and COLO320DM. Predicted or experimentally validated ecDNA breakpoints (marked in red) were derived from published work^7^ (Supplementary Table S1). scCirclehunter provided relatively precise breakpoint estimates, approaching the accuracy of AA. ATACamp, however, identified ecDNA fragments defined by integer multiples of bins, thereby limiting its precision for base-level breakpoint estimates.

### scCirclehunter uncovers ecDNA heterogeneity across GBM patients

Initially, we collected available scATAC-seq and snATAC-seq datasets, comprising 148 tumor samples from 10 cancers, to evaluate the frequency of ecDNA occurrence (Supplementary Tables S2, S3). Among these, ecDNA was identified in 75% (15/20) of the GBM samples from 13 patients (Supplementary Fig. S3a, b). To characterize ecDNA heterogeneity across patients, we examined the ecDNA in four adult GBM patients from the same batch. The results showed that three patients (GBM4250, GBM4275, and GBM4349) harbored ecEGFR amplifications, while one patient (GBM4218) did not. Notably, GBM4218 did not exhibit ecDNA amplification, while GBM4250 carried co-amplifications of *MDM2* and *CDK4* on ecDNA. Moreover, GBM4349 displayed ecMDM4 and ecPDGFRA amplifications (Supplementary Fig. S4a‒c). We then annotated cells from these four patients, integrating a total of 2879 cells, including 1895 malignant cells, 817 macrophages, 75 oligodendrocytes, and 92 T cells (Supplementary Fig. S4d). Interestingly, the GBM4218 sample without detecting ecDNA, contributed the majority of macrophages (472/817) and T cells (84/92) (Supplementary Fig. S4e)^12,23^. We compared the chromatin profiles of malignant cells from ecDNA-negative (ecDNA^‒^) GBM4218 and the other three ecDNA-positive (ecDNA^+^) patients. Pan-cancer analysis has identified 11 tumor-upregulated signatures and 7 immune-downregulated signatures associated with ecDNA^+^ samples^23^. We found that 8 out of 11 tumor features were significantly upregulated in the tumor cells from three ecDNA^+^ patients, including DNA repair, cell cycle, and *HOX* gene signatures. In contrast, 4 out of 7 immune features scored significantly higher in the tumor cells from the non-ecDNA GBM4218 patient. These findings suggest that GBM patients with ecDNA amplifications exhibit typical features associated with ecDNA presence (Supplementary Fig. S5c).

**Fig. 3:**
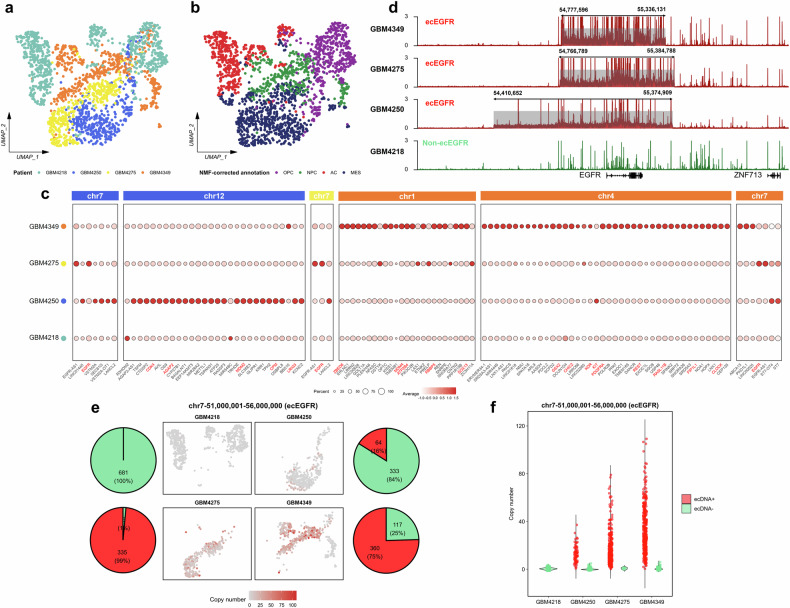
Heterogeneity of ecDNAs across different GBM patients. **a**, **b** UMAP projection of scATAC-seq data for malignant cells from four GBM patients, colored by patient (**a**) or cellular state (**b**). **c** Dot plot showing the GeneScore of ecDNA-carrying genes in malignant cells from the corresponding GBM patients. **d** Normalized accessible signals of malignant cells from four adult GBM patients in the ecEGFR region. Red tracks indicated patients carrying ecDNA, green tracks represented those without ecEGFR, and gray-shaded areas highlighted the ecEGFR regions. **e** UMAP plot illustrating the copy number of the ecEGFR region in malignant cells from each GBM patient. Pie chart showing the proportion of malignant cells predicted to carry ecEGFR. **f** Violin plot comparing the copy number of the ecEGFR region between cells with and without ecEGFR in each GBM patient. Statistical significance was assessed using a two-sided Wilcoxon test. ns, no significance; *P < 0.05; **P < 0.01; ***P < 0.001; ****P < 0.0001.

Previous scRNA-seq analysis on GBM defined four major tumor cellular states, including neural progenitor-like (NPC-like) cells, oligodendrocyte progenitor-like (OPC-like) cells, astrocyte-like (AC-like) cells and mesenchymal-like (MES-like) cells^14^. Based on scoring for these four cellular states, we employed nonnegative matrix factorization (NMF) to optimize the cell state annotations (Supplementary Fig. S4f). The final classification revealed that in GBM4218, the dominant states were AC-like and OPC-like, whereas in GBM4250 and GBM4275, the MES-like state was most prevalent. In contrast, GBM4349 predominantly consisted of cells exhibiting the OPC-like and NPC-like states (Fig. 3a, b; Supplementary Fig. S4g).

It has been demonstrated that ecDNA regions exhibit increased chromatin accessibility^7^. To further explore this, we analyzed the GeneScore of ecDNA-associated genes (ecGenes) across four patients. The results revealed that ecGenes generally show higher accessibility in the corresponding patients, suggesting that these genes may be more highly expressed compared to non-ecDNA patients, consistent with known features of ecDNA (Fig. 3c; Supplementary Fig. S5a). Specifically, the regions with elevated accessibility in the three GBM patients carrying ecEGFR completely overlapped with the ecDNA amplification regions we predicted (Fig. 3d). Furthermore, we found that the chromatin accessibility driven by ecDNA was distributed across different cellular states in each sample, with notable patient-specific variation (Supplementary Fig. S5a). For example, AC state cells did not display the highest *EGFR* accessibility, as these cells predominantly originated from the GBM4218 patient, which lacks the ecEGFR amplification. In contrast, the NPC state in GBM4349 and the MES state in GBM4275, both of which carry ecEGFR, exhibited higher accessibility at the *EGFR* locus. Notably, MES cells in GBM4275 showed higher accessibility, while MES cells in GBM4250, which had a lower proportion of cells carrying ecEGFR, exhibited lower *EGFR* accessibility signals (Fig. 3e; Supplementary Fig. S5a). These findings underscore the heterogeneity of ecDNA and suggest that it may be present in various cellular states, rather than being confined to a single state. This observation is consistent with previous studies that have linked ecDNA to multiple GBM cellular states^16,24^. Moreover, these results imply that, while ecDNA-driven gene accessibility spans different cellular states, transcriptional regulation may dictate the expression of specific ecGenes, such as *EGFR* and *PDGFRA*, in a manner dependent on the cell state.

We further examined the differences in ecDNA carrying the same oncogene across various GBM patients. Specifically, the ecEGFR in patient GBM4250 consists of a single 964 kb segment, whereas both GBM4275 and GBM4349 harbor ecEGFR with multiple rearranged segments (Supplementary Fig. S4b, c). Notably, we found that the ecEGFR regions coincide with the boundaries of continuous, highly accessible chromatin regions, suggesting that ecDNA is the primary driver of the increased accessibility signals within the region. Using the described method, we identified the cells carrying ecEGFR in each sample and applied a previously published approach to estimate the copy number of the ecEGFR region (chr7:51,000,001‒56,000,000)^5^. Our results revealed significant differences in the proportion of cells carrying ecEGFR across the three patients. In GBM4275, nearly all malignant cells contained ecEGFR, whereas in GBM4349, approximately 75% of the cells harbored ecEGFR. In contrast, only 16% of the cells in GBM4250 carried ecEGFR. The proportion of cells carrying ecEGFR was consistent with the number of cells identified by the method as having high-copy *EGFR* (Fig. 3e). Previous studies have shown that the unequal segregation of ecDNA can result in the accumulation of large numbers of ecDNAs in specific cells^25^. Subsequent studies revealed considerable variation in the number of ecDNA copies present in individual cells, ranging from just a few to over one hundred copies^2,26^. In our analysis of three ecDNA^+^ GBM patients, we observed significant heterogeneity in ecDNA copy numbers among cells. For instance, ecEGFR-positive cells in GBM4250 contained about 40 copies at most, while cells from GBM4275 carried as many as 80 copies, and those from GBM4349 harbored more than 100 ecEGFR copies (Fig. 3f). These findings highlight the considerable disparity in ecDNA copy numbers between cells and demonstrate that ecDNA accumulation results in a small subset of cells carrying exceptionally high copy numbers, consistent with previous reports^2,26^. Additionally, nearly all malignant cells from GBM4349 contained ecMDM4, with some cells also exhibiting high ecPDGFRA copies. However, nearly all malignant cells from GBM4250 displayed high copy numbers within the ecMDM2 region (Supplementary Fig. S5b). Overall, the widespread distribution of ecDNA copies within each patient underscores the substantial heterogeneity of ecDNA across different cells.

### Mutual exclusion, coexistence, and differentiation of distinct ecDNAs within a GBM patient

**Fig. 4:**
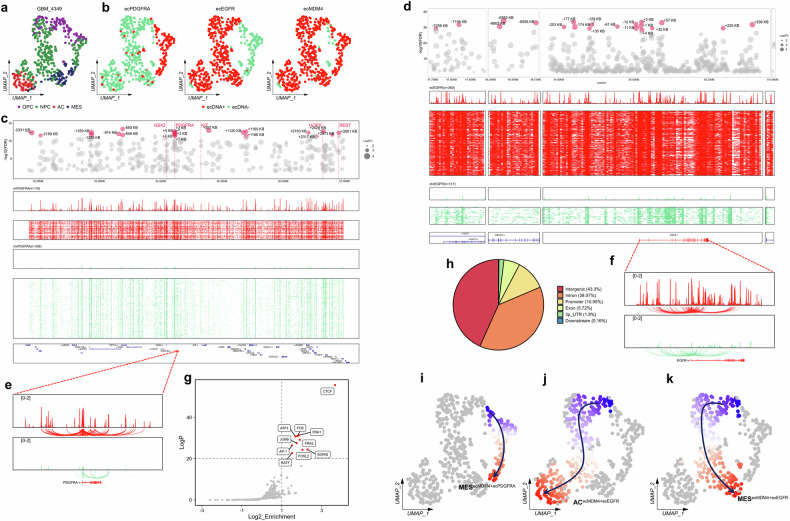
Associations between multiple ecDNAs within the GBM4349 patient. **a** UMAP projection of scATAC-seq data for 477 malignant cells from the GBM4349 patient, with cellular state annotation. **b** Distribution of malignant cells predicted to carry ecPDGFRA, ecEGFR, and ecMDM4. Malignant cells carrying ecDNA versus those without. Top to bottom, differentially accessible peaks within the ecDNA region for cells carrying ecDNA compared to those without, with FDR < 0.05 and log_2_FC > 0.5 retained. **c**, **d** The top 20 peaks with the highest log_2_FC were identified, and the distances to the *PDGFRA* (**c**)/*EGFR* (**d**) promoters were calculated and annotated. The overall normalized accessibility signal within the ecDNA region for the ecDNA^+^ cluster. Bigwig signals for each ecDNA^+^ cell within the ecDNA region. The overall normalized accessibility signal within the ecDNA region for the ecDNA^‒^ cluster. Bigwig signals for each ecDNA^‒^ cell within the ecDNA region. Gene annotations are provided. **e**, **f** Co-accessibility-based prediction of promoter‒enhancer interactions upstream and downstream of the *PDGFRA* (**e**)/*EGFR* (**f**) promoter for the ecDNA and non-ecDNA clusters. **g** Enrichment of motifs in differential peaks within the ecPDGFRA region in the ecPDGFRA cluster. **h** Genomic annotation of differential peaks within the ecEGFR region in the ecEGFR cluster. **i, j, k** Trajectories inferred for malignant cells in the GBM4349 patient.

There may be a potential relationship between multiple types of ecDNA within the patient. In the GBM4349 patient, we observed the co-existence of three types of ecDNA, namely ecMDM4, ecEGFR, and ecPDGFRA (Fig. 4a, b). Notably, ecMDM4 was present in most malignant cells and co-existed with either ecEGFR or ecPDGFRA. Specifically, 70.4% (336/477) of malignant cells were ecEGFR + ecMDM4, while 18% (86/477) were ecPDGFRA + ecMDM4 (Fig. 4a; Supplementary Fig. S6a‒d). However, ecEGFR and ecPDGFRA exhibited mutually exclusive patterns, consistent with previous findings^16,27,28^. Furthermore, almost no AC state cells were observed carrying ecPDGFRA. The distribution of ecDNA across different cellular states in this patient also displayed biased patterns. The proportion of cells carrying ecEGFR was highest in the AC state, while ecPDGFRA-harboring cells were most abundant in the OPC state. This suggests a potential association between specific ecDNA types and cellular states (Supplementary Fig. S6e, f). To further explore the relationship between ecDNA and GBM cellular states, we utilized publicly available single-cell data to deconvolute cell types from the TCGA datasets (Supplementary Fig. S6g). The ecDNA has been detected based on the corresponding whole-genome sequencing (WGS) data from the same patient^12^. We focused on three amplified genes associated with cellular states, including *PDGFRA*, *EGFR*, and *CDK4*^14^. Our analysis revealed that the proportion of AC state cells in patients with ecEGFR (*n* = 15) was significantly higher compared to patients with linear *EGFR* amplification (*n* = 10) and those without *EGFR* amplification (*n* = 22), while the proportion of OPC state cells was lower in the ecEGFR group than in non-ecEGFR patients. In contrast, ecCDK4 patients (*n* = 7) exhibited a lower proportion of MES state cells and a higher proportion of OPC/NPC state cells compared to non-ecCDK4 patients (*n* = 34). No significant trends were observed in the ecPDGFRA group (*n* = 3), likely due to the limited cohort (Supplementary Fig. S6h‒j). In summary, these findings suggest that ecEGFR and ecCDK4 may be associated with higher proportions of AC and OPC/NPC cellular states, respectively.

To compare the activation of regulatory elements within ecDNA regions at the single-cell level, we analyzed the accessibility signals and differential peaks between ecDNA^+^ and ecDNA^‒^ cells. The top 20 differential peaks with the highest fold-change were highlighted in red, and the linear distance to the promoters of key genes, including *EGFR*, *PDGFRA*, and *MDM4*, was calculated (Fig. 4c, d; Supplementary Fig. S6k). Compared to ecDNA^‒^ cells, ecDNA^+^ cells exhibited a globally enhanced open chromatin signal within the ecDNA regions. Notably, in the single-segment ecPDGFRA, promoters of key genes such as *GSX2*, *PDGFRA*, *HOPX*, and *REST* showed significantly increased accessibility, while in the multi-segment ecEGFR, a broad enhancement of open chromatin signals was observed across the entire region. Based on enhancer‒promoter predictions from scATAC-seq, more activated regulatory element signals were detected flanking the *PDGFRA* and *EGFR* promoters in ecDNA^+^ cells (Fig. 4e, f). We further annotated the differential peaks within the ecPDGFRA and ecEGFR regions and identified enriched transcription factor (TF) binding motifs. In the ecPDGFRA region, the differential peaks were highly enriched in both the promoter (39.14%) and intron (37.31%) regions. Motif enrichment analysis using HOMER revealed that the ecPDGFRA region was particularly enriched for *AP-1* family motifs, including *FOSL2* and *JUNB* (Fig. 4g), and TF activity analysis confirmed the enrichment of *AP-1* motif in MES cells (Supplementary Fig. S7a). The *AP-1* family is known to play a critical role in promoting a mesenchymal state^29^. In contrast, the ecEGFR region was predominantly enriched in intergenic (43.3%) and intron (38.07%) regions (Fig. 4h), and studies have confirmed the presence of super-enhancers within the ecEGFR region to regulate global transcription^6^.

Previous studies have shown that TFs carried by ecDNA can modulate the expression of target genes, driving tumor heterogeneity^2,8^. In the case of ecPDGFRA, the TF GSX2 is carried by the ecDNA, and its promoter region exhibits increased accessibility. Transcription factor deviation analysis further revealed that cells containing ecPDGFRA also exhibit high *FOXG1* activity (Supplementary Fig. S7b, c). *FOXG1* is a TF-coding gene that regulates the development, proliferation, and maintenance of cellular stemness^30^. STRING analysis further supported the hypothesis that GSX2 may regulate *FOXG1* expression (Supplementary Fig. S7d). To validate the relationship between ecGSX2 and *FOXG1*, we performed a differential analysis using RNA-seq data from the TCGA database. The results revealed that *FOXG1* expression was significantly higher in the ecGSX2 group compared to the non-amplified group, whereas no such trend was observed in the linear GSX2 amplification group (Supplementary Fig. S7e). Additionally, gene set enrichment analysis (GSEA) analysis indicated that FOXG1 target genes were markedly upregulated in the ecGSX2 group (*P* = 0.026) (Supplementary Fig. S7f). These findings suggest that ecGSX2 may employ a distinct mechanism to regulate *FOXG1*, which is not observed in the case of linear *GSX2* amplification. Collectively, our analysis provides preliminary insights into the regulatory mechanisms of ecDNA and highlights the potential role of ecDNA-associated TFs in promoting tumor heterogeneity.

Due to the mutual exclusivity and co-existence of different types of ecDNA, we hypothesize that they may correspond to distinct trajectories. To investigate this, we first performed unsupervised trajectory inference using monocle3. Considering that most cells harbor ecMDM4, we hypothesized that cells lacking this ecDNA could represent the root state. Indeed, the largest proportion of these cells was in the OPC state (48.2%), which is consistent with previous studies that used OPC/NPC states as the root cells^31^ (Supplementary Fig. S7g‒j). In summary, based on these findings, we identified three potential trajectories in the GBM4349 sample (Fig. 4i‒k). Trajectory 1 represents differentiation from OPC/NPC cells towards the MES state, with the terminal state being MES cells that carry both ecMDM4 and ecPDGFRA. Trajectories 2 and 3 lead to terminal states of AC and MES cells, respectively, with cells at the endpoints of these trajectories tending to carry both ecMDM4 and ecEGFR. This suggests that different types of ecDNA may be associated with distinct trajectories. Furthermore, the ecPDGFRA region is enriched with binding motifs for the AP-1 TF family, which promotes mesenchymal transition (Fig. 4g). This finding may be linked to the terminal MES state in the ecPDGFRA-associated trajectory.

### Comprehensive analysis of scRNA-seq and scATAC-seq data reveals diverse oncogenic mechanisms driven by ecNR2E1

**Fig. 5:**
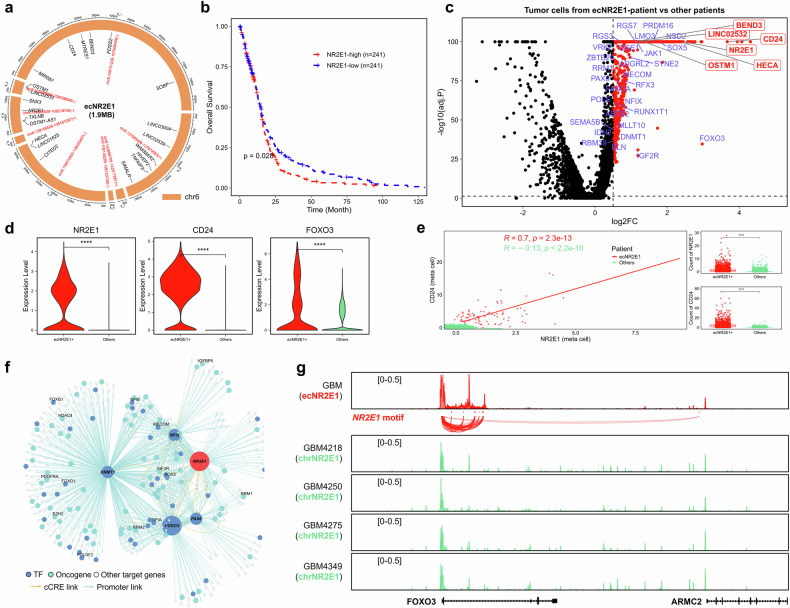
Diverse oncogenic mechanisms induced by ecNR2E1. **a** Reconstruction of ecNR2E1 circular amplification based on scATAC data. **b** Kaplan–Meier analysis of overall survival in TCGA-GBM patients separated by *NR2E1* expression. **c** Volcano plot showing differentially expressed genes from ecNR2E1 patient-derived malignant cells compared to malignant cells from other patients, with fold change (FC) and adjusted *P* value (adj.*P*) calculated using the FindAllMarkers function. Genes with Log_2_FC > 0.5 and adj.*P* < 0.05 were considered upregulated. Differentially expressed TFs and oncogenes in the constructed ecNR2E1 network were highlighted in blue, while differentially expressed genes carried by ecNR2E1 were marked in red. **d** Violin plots showing the expression of *CD24*, *NR2E1*, and *FOXO3* in malignant cells from ecNR2E1 and other patients. Statistical significance was assessed using a two-sided Wilcoxon test. **e** Meta-cells of ecNR2E1 and other patient-derived malignant cells were constructed, and the correlation between *CD24* and *NR2E1* counts was calculated using the Pearson method. Boxplots showing *CD24* and *NR2E1* counts for each group. Statistical significance was assessed using a two-sided Wilcoxon test. ns, no significance; *P < 0.05; **P < 0.01; ***P < 0.001; ****P < 0.0001. **f** TF regulatory network depicting the candidate target genes for *NR2E1* and *FOXO3* in malignant cells from the ecNR2E1 patient. The network included 511 of the 1045 upregulated genes identified in ecNR2E1 patient-derived malignant cells. **g** Enhancer activity analysis identified candidate enhancers that correlated with *FOXO3* expression, contained *NR2E1* recognition motifs and were differentially active between malignant cells from ecNR2E1 and non-ecNR2E1 patients.

Previous scRNA-seq studies have shown that the heterogeneity of the MYC family carried by ecDNA across different tumor cells significantly influences cellular transcriptional programs in neuroblastoma, small-cell lung cancer, and pancreatic ductal adenocarcinoma^2,8,9^. In this study, we analyzed paired scATAC + scRNA-seq data from a GBM patient carrying an ecDNA comprising eight segments on chromosome 6, spanning 1.9 Mb (Fig. 5a), with high sequencing coverage (Supplementary Fig. S8a, b). This ecDNA harbors the TF NR2E1 (TLX), which has been implicated in tumor immune suppression and immune cell infiltration^32^. Survival analysis revealed that high expression of *NR2E1* is associated with poor prognosis in GBM patients (Fig. 5b). Additionally, this ecDNA carries the *CD24*, whose high expression has been linked to tumor cell growth, metastasis, and resistance to apoptosis. CD24 inhibits macrophage activity through interaction with SIGLEC10, promoting immune evasion in tumors, and thus represents a potential target for GBM immunotherapy^33–35^. Overall, this ecDNA reveals several mechanisms that lead to tumor progression and treatment resistance.

To further investigate the mechanisms by which ecNR2E1 regulates downstream genes, we integrated malignant cells from snRNA-seq data of multiple GBM patients^29^. Differential expression analysis revealed significant upregulation of both *NR2E1* and *CD24* in the ecNR2E1-positive patient (Supplementary Fig. S8c), with log_2_ fold changes of 3.6 for *CD24* and 2.4 for *NR2E1* (Fig. 5c, d; Supplementary Table S6). scCirclehunter predicted that most malignant cells from the ecNR2E1-positive patient harbor ecNR2E1 (Supplementary Fig. S8d), and a significant positive correlation was observed between the counts of *NR2E1* and *CD24* in these ecNR2E1 patient-derived malignant metacells (Pearson correlation = 0.7) (Fig. 5e). In contrast, no such correlation was found in other patients, and TCGA data further revealed a weak negative correlation between these two genes across RNA-seq data from 166 GBM patients (Supplementary Fig. S8e). These findings suggest that the presence of ecDNA aberrantly activates its carried genes, driving substantial transcriptional heterogeneity among different patients.

To explore whether ecNR2E1 regulates downstream target genes and forms a novel regulatory network similar to ecMYC, we employed a published method to identify NR2E1 target genes and candidate *cis*-regulatory elements (cCREs)^36^. These cCREs are accessible in tumor cells and capable of binding transcription factors. Based on this, we constructed a regulatory network for 1045 upregulated genes in the ecNR2E1-positive patient. Key NR2E1 target genes include *FOXO3*, *NFIX*, *PAX6*, and *DNMT1*, which, along with their downstream targets, account for 511 of total 1045 upregulated genes. Further analysis showed that the upregulated genes in ecNR2E1 patient were enriched in pathways related to stem cell maintenance and proliferation (Supplementary Fig. S8f). Notably, NR2E1 regulates several genes associated with GBM proliferation and invasion, including the transcription factor-coding genes *FOXO3*, *PAX6*, *NFIX*, *SOX5*, and the oncogene *IGF2R* (Fig. 5f)^16,37–39^. We found that NR2E1 binds to enhancers to regulate *FOXO3* expression, while in other GBM patients lacking ecNR2E1, the enhancers bound by NR2E1 remain inactive in malignant cells (Fig. 5g). *FOXO3* overexpression promotes GBM invasion and correlates with tumor progression^37^, and it is significantly upregulated in malignant cells from ecNR2E1-positive patient (log_2_FC = 2.9). *FOXO3* may also be a potential target gene for TFs, including PAX6, NFIX, SOX5, and PRDM16, which regulate *FOXO3* expression through enhancer binding, while DNMT1 and NFIX regulate *FOXO3* via promoter binding. All these genes are controlled by NR2E1 (Fig. 5f; Supplementary Table S7). TCGA data further validated the positive correlation between *NR2E1*, *PAX6*, *NFIX*, and *FOXO3*, supporting a regulatory mechanism through *FOXO3* (Supplementary Fig. S8g). These genes were all significantly upregulated in malignant cells from ecNR2E1-positive patient (Supplementary Fig. S8h). Lastly, we observed specific expression of *SIGLEC10* in immune cells from the ecNR2E1-positive patient, suggesting that the overexpression of *CD24* due to ecDNA may facilitate immune evasion in GBM (Supplementary Fig. S8i, j)^34^. Overall, we identified ecDNA carrying *NR2E1* and *CD24* and constructed a regulatory network for NR2E1 through paired scATAC + scRNA-seq data. Our findings indicate that the presence of ecDNA promotes GBM progression and tumor heterogeneity by harboring transcription factors and immune checkpoint genes.

### Multi-omics analysis reveals spatial heterogeneity of GBM-derived ecDNA

**Fig. 6:**
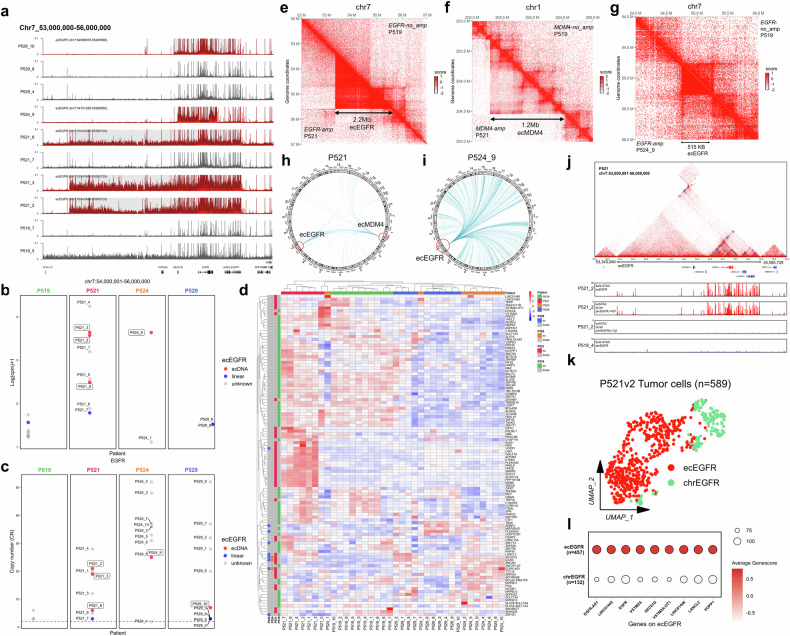
Spatial heterogeneity of ecDNA from four GBM patients. **a** The snATAC-seq signals of GBM patients, including P519, P521, P524, and P529, carry ecEGFR across multiple spatial regions. Red tracks indicated patients with ecEGFR, while gray tracks represented those without ecEGFR. Gray-shaded areas highlighted the ecEGFR regions. **b** Normalized bulk ATAC-seq signals from ecEGFR regions across distinct spatial regions in four patients. Red dots indicated the presence of ecEGFR as identified by scATAC-seq, blue dots indicated its absence, and gray dots indicated regions without corresponding scATAC-seq data. **c** Copy number of *EGFR* inferred from WES data across distinct spatial regions in four patients. **d** Heatmap showing the RNA expression of genes on ecDNAs across distinct spatial regions in four patients. ecGenes detected in each patient were annotated on the left. **e**‒**g** Hi-C maps showing the difference between ecDNA (left) and non-ecDNA (right) signals, including the 2.2 Mb-sized ecEGFR from P521 (**e**), the 1.2 Mb-sized ecMDM4 from P521 (**f**), and the 515 kb-sized ecEGFR from P524_9 (**g**), with P519 used as the control. **h** Circos plot showing *trans*-interaction profiles of ecDNA regions in P521. **i** Circos plot showing *trans*-interaction profiles of ecDNA regions in P524_9. **j** Hi-C maps showing the regulations within the P521 ecEGFR region, with circles representing predicted loops. From top to bottom: gene annotations, bulk ATAC-seq signal from P521_2, normalized scATAC-seq signal from ecEGFR-positive cells of P521_2, normalized scATAC-seq signal from non-ecEGFR cells of P521_2, and bulk ATAC-seq signal from P519_4 (used as control) without ecEGFR. **k** Dimensionality reduction of cells from P521_2 based on scATAC-seq data, colored by predicted ecEGFR state. **l** Dotplot showing the GeneScore of ecEGFR-carrying genes in P521_2 cells based on scATAC-seq data, grouped by ecEGFR state.

Recent studies have provided a comprehensive view of the tumor spatial landscape in GBM patients^28^. In this study, we sought to investigate the variability of ecDNA across different spatial regions within individual patients and explore the regulatory mechanisms mediated by ecDNA. Using scATAC-seq data, we identified ecDNA in multiple regions from four patients: P519, P521, P524, and P529. In the P521 patient, we detected a 2.2 Mb ecEGFR and a 1.2 Mb ecMDM4, while P524 carried a 515 kb ecEGFR and P529 had a 796 kb ecEGFR (Fig. 6a; Supplementary Table S4). Our results revealed that ecDNA is not uniformly distributed across different regions within the same patient. For example, the P521_7 sample showed only a weak signal in the ecEGFR region, and no ecDNA was detected. Similarly, under identical detection conditions, ecEGFR was absent in both the P529_4 and P529_8 samples. Further analysis of corresponding whole-exome sequencing (WES) and ATAC-seq data revealed that regions with detectable ecEGFR also exhibited higher *EGFR* copy numbers compared to regions where ecEGFR was absent. Notably, in the P521_7 sample, where ecEGFR was not detected, both *EGFR* copy number and chromatin accessibility signals were the lowest among all eight samples from this patient (Fig. 6b, c; Supplementary Fig. S9a, b). In addition, we analyzed the gene expression associated with ecDNA in the P521_3 sample and found that these genes were among the highest expressed. In contrast, genes in other samples lacking ecDNA did not show this expression pattern (Supplementary Fig. S9c, d). We also examined RNA expression of ecDNA-carrying genes across different regions from four patients. While expression profiles were relatively consistent across regions within the same patient, significant heterogeneity in ecGene expression was observed, particularly in samples from P524 (Fig. 6d). Hi-C interaction analysis further revealed that chromatin regions with frequent interactions coincided with the boundaries of ecDNA, such as the ecEGFR and ecMDM4 regions in P521 (Fig. 6e, f), and the ecEGFR region in P524_9 (Fig. 6g). This spatial heterogeneity in chromatin interactions was also evident in P529, where P529_1 displayed strong interaction signals within the ecEGFR region, whereas P529_8 exhibited a pattern resembling that of patient P519 without ecEGFR (Supplementary Fig. S9e, f). Together, these results highlighted substantial heterogeneity in ecDNA distribution, chromatin interactions, and gene expression across different tumor regions within the same patient.

We further investigated the *trans*-regulatory function of ecDNA through Hi-C analysis^5,6,40^, revealing extensive genome-wide interactions in both P521 and P524_9 ecDNA regions, particularly involving the ecEGFR region (Fig. 6h, i). We filtered the Hi-C interaction data from P521 and P524_9 to identify genes interacting with the ecEGFR region across the genome. Hypergeometric testing of these genes, comparing them to differentially expressed genes in P521 (ecEGFR^+^) and P524 (ecEGFR^+^) relative to P519 (ecEGFR^‒^), revealed significant associations (*P* values of 1e‒5 for P521 and 1e‒50 for P524_9). This analysis suggested that genes regulated by the ecEGFR tend to be highly expressed, particularly in P524_9, indicating that ecEGFR may facilitate widespread transcriptional enhancement (Supplementary Fig. S9g, h). Furthermore, in P521, we observed frequent interactions between ecEGFR and ecMDM4 (Fig. 6h). This interaction remained the most statistically significant even after adjusting for copy number effects (adj.*P* = 0, Supplementary Fig. S9i), suggesting potential cooperation between distinct ecDNAs^5,6,40^.

By analyzing Hi-C data, we identified loops that overlap with ecDNA regions. Notably, we identified a chromatin loop anchored by the *EGFR* promoter spanning a distance of 400 kb. (Fig. 6j). In the ecMDM4 region, we observed multiple loops, each anchored at one end by the promoters of genes, including *ZBED6* (1.2 Mb apart), and *FMOD* (360 kb apart) (Supplementary Fig. S10a). These loops suggest the potential for enhancer-hijacking events mediated by the ecDNA. Consistently, chromatin loops were detected at the breakpoints of both ecEGFR and ecMDM4. We analyzed the bulk ATAC-seq signal profile for P521_2 and classified 589 malignant cells from this sample into two groups: 457 ecEGFR cells and 132 chrEGFR cells (Fig. 6k, l). We then generated scATAC-seq signals for two groups. The signal from the ecEGFR group closely mirrored the bulk ATAC-seq profile of P521_2, whereas the chrEGFR group lacked the signal specific to the ecEGFR region. The chromatin accessibility profile of the chrEGFR cells closely resembled that observed in the bulk ATAC-seq data for P519, a control sample without ecEGFR amplification. Similar findings were observed in the ecMDM4 region of P521_3, further distinguishing ecDNA from non-ecDNA signals (Supplementary Fig. S10b, c). These results support our approach in distinguishing ecDNA^+^ cells from those with non-ecDNA, highlighting the distinctive chromatin accessibility profiles associated with ecDNA amplification events.

Finally, we aimed to demonstrate that scATAC-seq could predict regulations within ecDNA regions, and we divided the genome into 1 Mb bins and quantified the number of regulatory interactions inferred by scATAC-seq within each bin. Our analysis revealed that the most densely regulated regions of the genome were ecMDM4 and ecEGFR (Supplementary Fig. S10d). We then focused on the ecEGFR region located on chromosome 7 (chr7: 53,000,000‒56,000,000) and analyzed the regulatory interactions within this region across different scATAC-seq samples. Notably, we observed a sharp increase in the number of regulations between ecEGFR and adjacent bins, particularly in the regions of 53.4‒55.6 Mb for P521, 54.8‒55.3 Mb for P524, and 54.8‒55.5 Mb for P529, which all correspond to ecEGFR events. These regions showed significantly more regulatory activity compared to regions lacking ecDNA. Importantly, the signal from P529_8, which does not carry ecEGFR, displayed a smooth pattern with no significant fluctuations in the corresponding genomic region (Supplementary Fig. S10e). This observation is consistent with the idea that ecDNA amplifications can drive localized and enhanced regulatory interactions. These findings underscore the power of scATAC-seq to predict regulatory interactions within ecDNA regions, and the results were in strong agreement with the high-frequency interaction signals observed in Hi-C analysis.

### ecDNA and mitochondrial transfer

**Fig. 7:**
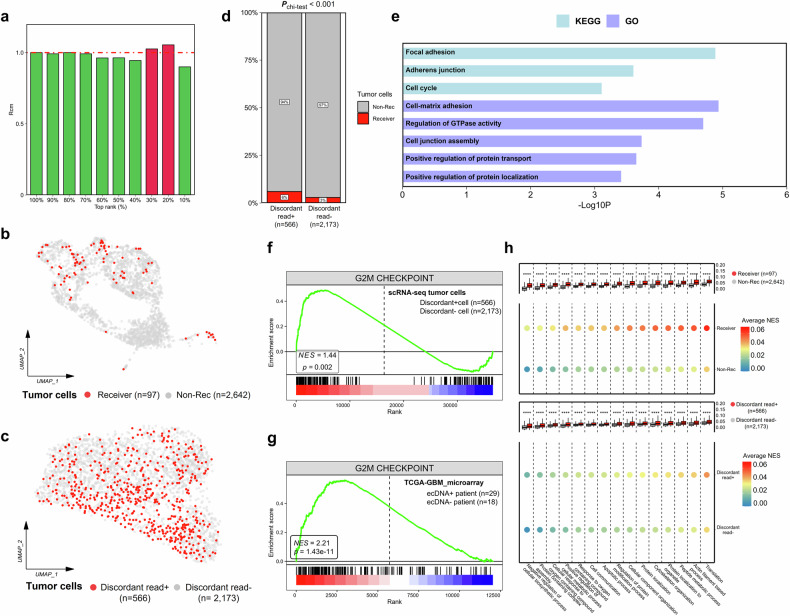
Commonalities between malignant cells carrying ecDNA and mitochondria hijacking. **a** Significance estimation of the number of positive calls reported by MERCI for a GBM patient with paired scATAC-seq + scRNA-seq data. **b** UMAP plot showing the dimensionality reduction result of malignant cells (*n* = 2739) from scRNA-seq data, without regressing the percentage of mitochondrial reads. Malignant cells predicted to hijack mitochondria were marked in red. **c** UMAP plot showing the dimensionality reduction result of malignant cells (*n* = 2739) from scATAC-seq data. Malignant cells carrying discordant reads at the detected ecDNA breakpoints are marked in red. **d** Cells carrying discordant reads in the mitochondrial transfer phenotype. The *P*-value estimated using the *χ*^2^ test indicated the association between cells carrying ecDNA and hijacking MT. **e** Pathways enriched for upregulated genes in malignant cells that hijacked MT compared to those that did not, in the GBM patient. GSEA plot of genes involved G2M checkpoint. Grouping of malignant cells from the GBM patient based on the presence of discordant reads (**f**). Grouping of 47 TCGA-GBM patients based on ecDNA detection (**g**). **h** scGSVA analyzed MT transfer-related pathways in mitochondrial transfer and ecDNA groups.

A pan-cancer study has demonstrated that ecDNA-containing tumors exhibit upregulation of three key biological processes, including DNA damage repair, cell cycle, and cell proliferation (e.g., HOX family, SHCBP1), compared to non-ecDNA tumors^23^. This suggests that ecDNA-bearing tumor cells may be in a more active cell cycle and proliferative state than non-ecDNA cells. Accordingly, we hypothesize that ecDNA-containing tumor cells are more likely to modulate mitochondrial function to generate increased energy, thereby supporting rapid proliferation. Recent research has indicated that tumor cells provide themselves with additional energy by hijacking mitochondria (MT) from non-malignant cells^41,42^, and we applied a similar analysis to a GBM sample with paired scATAC + scRNA-seq data. Since macrophages are an essential component of the GBM immune microenvironment, we categorized non-malignant cells, including macrophages, as mitochondrial donors, while malignant cells were considered mitochondrial recipients. We utilized MERCI, a deconvolution approach based on scRNA-seq, to identify mitochondrial recipient malignant cells in tumor samples based on mitochondrial DNA variants and mitochondrial gene expression. MERCI analysis of the paired GBM sample revealed the presence of mitochondrial hijacking (Fig. 7a, b). Additionally, ecDNAs were detected in the sample. Cells with discordant reads at identified ecDNA breakpoints were classified as high-confidence ecDNA^+^ cells (Discordant+, Fig. 7c). We observed a higher proportion of tumor cells hijacking MT in the Discordant^+^ group (*χ*^2^ test, *P* < 0.001), suggesting that ecDNA^+^ cells may have a greater prevalence of mitochondrial hijacking (Fig. 7d). To validate this, we incorporated an additional cohort involving 19 clear cell renal cell carcinoma (ccRCC) samples^43^, each of which had both scATAC-seq and scRNA-seq data from the same patient, although these were not paired data from the same individual cells. First, we used scATAC-seq data to predict whether the patient harbored ecDNA (Supplementary Tables S2, S3). Subsequently, we applied MERCI, a method based on scRNA-seq, to infer whether mitochondrial hijacking occurred in the same patient and to estimate the proportion of tumor cells involved in mitochondrial hijacking. To ensure the reproducibility of our results, we utilized Cancer-Finder^44^, a deep learning-based tool for classifying malignant cells, to distinguish between malignant and non-malignant cells in each ccRCC patient. Non-malignant cells were considered as mitochondrial donors, while malignant cells were regarded as mitochondrial recipients. We then selected the percentage with the highest RCM values to estimate the proportion of malignant cells hijacking MT (Supplementary Figs. S11a, b). Our results indicated that three out of six ecDNA^+^ ccRCC patients exhibited mitochondrial hijacking (3/6), while only 2 out of 13 ecDNA^‒^ ccRCC patients showed mitochondrial hijacking (2/13) (Supplementary Fig. S11c). Furthermore, data from ecDNA^+^ ccRCC patients supported a higher proportion of tumor cells with mitochondrial hijacking compared to ecDNA^‒^ patients (Supplementary Fig. S11d). In summary, these results indicate that ecDNA may be associated with a higher incidence of mitochondrial transfer.

We further analyzed the phenotypes of ecDNA^+^ tumor cells and mitochondrial hijacking-positive (MT^+^) tumor cells in this GBM patient. Pathway analysis of the upregulated genes in MT^+^ cells revealed that the most significantly enriched pathways were related to cell adhesion, protein localization and transport, and the cell cycle (Fig. 7e). These pathways were consistent with previously reported phenotypes of MT^+^ tumor cells, thus supporting the validity of our findings^41^. Next, we compared the differential hallmark signaling pathways between Discordant^+^ and Discordant^‒^ tumor cells and identified the G2/M checkpoint as the most significantly enriched gene set (NES = 1.44, *P* < 0.01, Fig. 7f). This gene set was also significantly upregulated in the ecDNA^+^ group of TCGA-GBM (NES = 2.21, *P* < 0.001, Fig. 7g), consistent with the general features of ecDNA observed in pan-cancer analysis^23^. These results collectively suggested that cell cycle dysregulation is a critical feature of GBM patients harboring ecDNA and may serve as a potential therapeutic target. Using single-cell Gene Set Variation Analysis (GSVA), we compared the significance of all Gene Ontology (GO) terms between Discordant^+^ and Discordant^‒^, as well as between MT^+^ and MT^‒^ cells. We found that features associated with mitochondrial transfer in tumor cells, such as the cytoskeleton, actin filaments, and pathways involved in nanotube formation, were significantly upregulated in the positive samples of both groups (Fig. 7h).

Overall, our results indicated that tumor samples exhibiting mitochondrial hijacking also showed activation of certain mitochondrial hijacking-related pathways in tumor cells harboring ecDNA, compared to control cells. This suggested an overlap in the cellular identity of these two conditions, with ecDNA^+^ samples potentially corresponding to a higher frequency of mitochondrial hijacking.

## Discussion

**Fig. 8:**
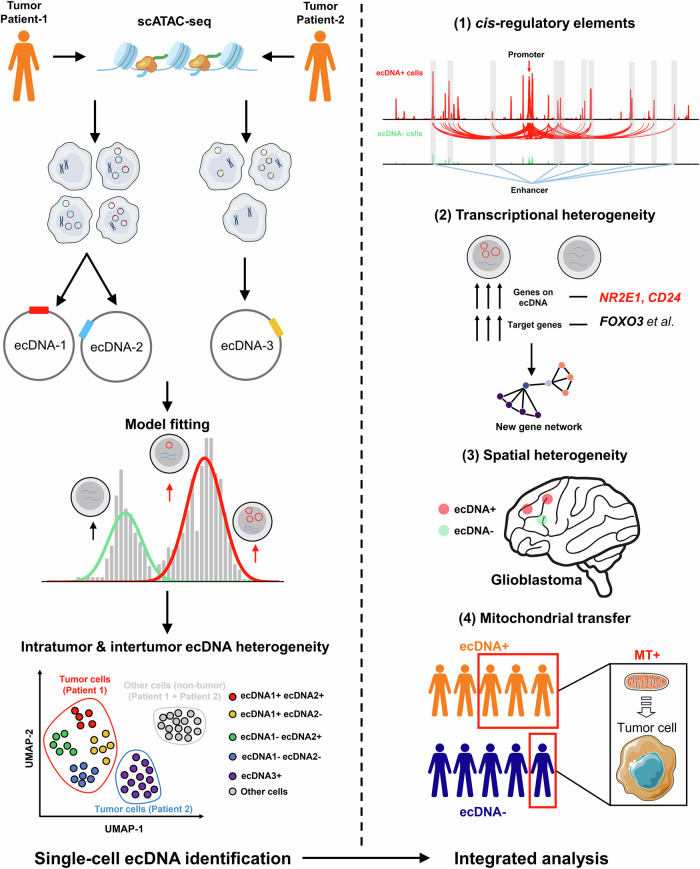
Graphical abstract of the study. Our study primarily comprises two main parts: identifying cells harboring specific ecDNA and centering on GBM for ecDNA analysis.

Since the discovery of ecDNA, researchers have been interested in its mechanisms that differ from those of linear chromosomes. Recently, advances in large-scale sequencing have made ecDNA a focus of cancer research^12,23^. Single-cell sequencing technology has become a mainstream and indispensable means for analyzing tumor heterogeneity and immune microenvironment^31^. Therefore, the integration of single-cell sequencing and ecDNA is very attractive, and preliminary attempts have been made^2,5,8,16,20,26,45–49^. Here, we describe scCirclehunter, a framework designed to identify and assign ecDNA to specific cell populations. Applying scCirclehunter to GBM, we identified frequently amplified genes on ecDNA and characterized intratumoral and intertumoral heterogeneity in ecDNA-bearing cells at single-cell resolution. Our analysis revealed that ecDNA exhibits considerable variation across different samples, not only in terms of the cancer-related genes it carries, but also in the structures, copy number, and proportion of cells containing ecDNA. Furthermore, ecDNA is present in a variety of cellular states in GBM, suggesting the existence of complex regulatory mechanisms controlling the expression of ecGenes across different cellular states. In addition, we integrated scRNA-seq data to examine ecDNA-mediated transcriptional alterations and the association between ecDNA and mitochondrial transfer. In our study, the existence of ecNR2E1 leads to the formation of a novel TF‒gene regulatory network, similar to the regulation of downstream targets by ecMYC, thereby driving tumorigenesis. Consequently, targeting ecDNA may provide more effective strategies for cancer treatments (Fig. 8)^32,33,37^.

Overall, scCirclehunter represents our meaningful attempt to study patient-derived ecDNA using single-cell sequencing. However, our method still has deficiencies, such as the natural disadvantages of scATAC-seq based on short-read sequencing in detecting structural variations compared with third-generation sequencing^10^. In addition, a major dilemma is that research on patient-derived ecDNA is relatively limited, especially lacking ecDNA patients with single-cell sequencing data. Although it is theoretically feasible to use a pseudo-bulk strategy to identify ecDNA in single-cell libraries when assigning ecDNA to specific cell populations, only a very small proportion of cells carry discordant reads supporting circular structures due to the sparsity of single-cell data. We have to choose a model to expand the potential ecDNA^+^ cell populations, which assumes that ecDNA is the main contributor to accessible chromatin signals in the corresponding regions of the genome in ecDNA patients. However, this strategy may not always be useful, especially when ecDNA and HSR coexist and transform into each other. We also considered using some classification methods, such as machine learning, but the core challenge is that we need labeled training data, and the single-cell data with ecDNA information is extremely limited, basically limited to several classic cell lines such as COLO320DM and GBM39, but the data of cell lines are far from enough to reflect the complex features of ecDNA in patients. For example, ecDNA in cell lines is generally high-copy, while low-copy ecDNA in patients may be the product of early amplification^10,50^. Moreover, ecDNA in cell lines often carries classic oncogenes, such as *MYC* and *EGFR*^5,7^, while the genes carried by ecDNA from patients are more diverse. Whether it is possible to train sufficiently accurate models based on single-cell data of cell lines for patient prediction requires more attempts.

Definitely, there are still some limitations in the experimental design and analysis of our study. Notably, the ability of our method in distinguishing between ecDNA- and HSR-amplified cells remains experimentally unvalidated. Furthermore, observed accessibility and genome-wide interaction increases within ecDNA regions could be influenced by elevated ecDNA copies, and correcting copy number would strengthen confidence in these findings. Interestingly, we observed upregulation of the G2M checkpoint in GBM patients harboring ecDNA, which aligns with a prior study demonstrating broad upregulation of cell cycle regulatory pathways, including mitotic/meiotic cell cycle, G1/S, and G2/M phases in ecDNA-containing tumors. Collectively, these observations suggest a possible trend whereby checkpoint activation resulting from ecDNA genomic instability coexists with cell cycle acceleration. Regarding the study design, we acknowledged that our analysis relied exclusively on public datasets and lacked patient-level experimental validation. Specifically, we did not conduct assays such as fluorescence in situ hybridization (FISH) to verify the concordance between computationally predicted ecDNA-bearing cells and their corresponding biological states, nor did we experimentally validate the association between ecDNA amplification and mitochondrial transfer. These limitations partially originate from the uncertainty in ecDNA detection rates and the complexity and heterogeneity of ecDNA within patients. Consequently, this defines a critical path for our future work by expanding patient cohorts and leveraging single-cell sequencing coupled with multi-dimensional data such as WGS to enhance the credibility of our conclusions.

Single-cell technologies, including SMOOTH-seq, scCircle-seq, and scEC&T-seq, have been developed to study circular DNA^45,46,49^, but these methods are often expensive, have low throughput, and are mainly designed to detect circular DNA rather than explore ecDNA regulation. Therefore, studies using these methods typically focus on cell lines with confirmed ecDNA, while the cellular composition in patients is more complex. Recently, scHi-C has been proposed to study ecDNA^24^, enabling the sequencing of thousands of cells in patients. However, since current ecDNA identification tools primarily rely on WGS, WES, ATAC-seq, and Circle-seq data, there is a need for specialized tools using Hi-C for ecDNA detection. Overall, the application of the aforementioned single-cell technologies is relatively specialized, but they have certain limitations when studying patient-derived ecDNA. In contrast, the combination of scATAC-seq and scRNA-seq has become common in studies of regulatory mechanisms across cell populations, with large-scale cohort sequencing completed^52^. Therefore, scCirclehunter offers an economical and feasible solution for understanding how cells with ecDNA drive heterogeneity and regulatory network activation at the single-cell level. Our methods hold promise for advancing ecDNA research in tumor diagnosis and treatment.

## Materials and methods

### Single-cell RNA and ATAC-seq data processing and analysis

scATAC-seq reads were aligned to the hg38 reference genome using cellranger-atac (10X Genomics, v.2.1.0), while paired scRNA-seq and scATAC-seq reads were processed with cellranger-arc (10X Genomics, v.2.0.2) to align to the hg38 reference genome. For scATAC-seq BAM files provided by EGA, bamtofastq (v.1.4.1) was used to convert them back to FASTQ format, followed by realignment to hg38 using cellranger-atac.

The ArchR R package (v.1.0.2) was used to analyze scATAC-seq data^11^. Low-quality cells were filtered based on the following criteria: peak region fragments > 1000, peak region fragments < 100,000, and TSS enrichment > 4. Additionally, only cell barcodes marked as “1” in the is__cell_barcode column of singlecell.csv (scATAC-seq) or in the is_cell column of per_barcode_metrics.csv (paired scATAC-seq + scRNA-seq) were retained, thereby ensuring that only cells identified as high quality by the 10X pipeline were included. Doublets were removed using ArchR. Dimensionality reduction of the ATAC-seq data was performed with iterative Latent Semantic Indexing (LSI) using the addIterativeLSI function, followed by UMAP embedding with the addUMAP function. Batch correction for multi-sample integration was applied using the addHarmony function. Gene activity was quantified by local chromatin accessibility using the addGeneScoreMatrix function. Differentially accessible peaks within ecDNA regions were identified using the getMarkerFeatures function to compare tumor cells in ecDNA^+^ and ecDNA^‒^ groups, filtering for differentially accessible elements in the ecDNA^+^ group based on FDR ≤ 0.05 and Log_2_FC ≥ 1. The genomic regions containing accessible chromatin peaks were annotated by ChIPseeker (v.1.36.0) with the UCSC database on hg38. Single-cell motif accessibility z-scores were computed with the cisBP motif database using addDeviationsMatrix function, and visualized with plotGroups and plotEmbedding functions. BigWig files for ecDNA^+/‒^ groups were generated using the getGroupBW function and visualized using the ggcoverage R package (v.1.4.0)^53^. Co-accessibility relationships within ecDNA regions were inferred using the addCoAccessibility function, accounting for the proximity between elements due to the circular structure of ecDNA by setting a maximum distance of 2 Mb and a correlation threshold of 0.8. The addTrajectory function was used to add supervised trajectories. The addModuleScore function was used to calculate the scores for gene sets. Gene sets associated with pathways that are upregulated and downregulated in ecDNA-containing tumors were collected from the published work^23^.

Seurat (v.4.1.0) R package was used to perform filtering, normalization, dimensionality reduction, clustering, and differential expression analysis for scRNA-seq data^54^. During the analysis of mitochondrial transfer, the percentage of mitochondrial reads was not regressed. For other analyses, the ScaleData function was used to regress both nCount_RNA and percent.MT. The batch effect across different samples was eliminated by the Harmony (v1.1.0) method^55^. Differential gene analysis was performed by the MAST method (“FindAllMarkers” function). Differentially expressed genes were identified with Bonferroni adjusted *P*-value < 0.05 and |avg_log_2_FC | > 0.5. We assessed the cell cycle phase of cells using the CellCycleScoring function of the Seurat R package with default parameter. The irGSEA R package (v.2.1.5) was employed to score the scRNA-seq data using the irGSEA.score function, with the UCell scoring method applied^56^.

### Simulation of ecDNA focal amplifications

To evaluate the performance in identifying ecDNA from scATAC-seq data using a pseudo-bulk strategy, we randomly generated 100 ecDNAs with the previously described method^10^. The sizes of the ecDNAs ranged from 500 kb to 10 Mb, with all segments randomly selected from the hg38 genome. Each segment was assigned a random ligation direction, and sequences were extracted from the genome accordingly. The sequences corresponding to the same ecDNA were ligated sequentially into one large circular sequence. Pair-end sequencing was simulated using ART with a read length of 50 bp^57^. The scATAC-seq data from the 10X PBMC1k sample were used as the background.

We simulated the bin-by-cell count matrix for ecDNA focal amplification using the simATAC package (v.1.0.0), following the published method^15,19^. First, we employed the simATAC package to learn parameters from the peak matrix of 10X PBMC1k normal cells, with the input consisting of 10 kb non-overlapping bins. The package then generated a matrix for 1000 cells with similar distributions, where the rows represented bins (denoted as ${{bin}}_{i}$) and the columns represented cells (denoted as ${{cell}}_{j}$), and estimated a Poisson mean for each entry, denoted as ${\lambda }_{{ij}}$. Subsequently, we randomly extracted genomic segments from the hg38 reference genome to simulate ecDNA. Due to the sparsity of scATAC-seq peaks, we retained only ecDNA regions with at least 3% overlap with PBMC1k peak regions. In total, 100 ecDNAs were simulated, ranging in size from 50 kb to 10 Mb, including ecDNAs composed of segments spanning multiple chromosomes as well as those from the same chromosome. Next, we generated a bin-by-cell copy number matrix ***C***, dividing the 1000 cells into 5 equal-sized groups, with ***m*** groups of cells designated as harboring a focal amplification. To simulate ecDNA focal amplification, we selected cells in group ***m*** and multiplied Poisson mean of entries in these cells that overlapped with ecDNA by a copy ratio, simulating varying levels of focal amplification, while all other elements of matrix ***C*** were set to 2, representing the normal diploid state. Finally, we generated a matrix ***X*** of the same size as ***C***, representing the corresponding scATAC-seq count matrix after incorporating focal amplification. For training, we used the 10X PBMC1k scATAC-seq data, setting the parameter sparse.fac to 0.5 when using simATAC package. The whole process could be summarized as:

$${{\boldsymbol{X}}}_{{ij}} \sim {Poisson}(0.5{{\boldsymbol{C}}}_{{ij}}{{\boldsymbol{\lambda }}}_{{ij}})$$

Among them, ***m*** varied from {1, 2, 3, 4}, and the copy ratio of focal amplifications varied from {3, 5, 10, 20}. We repeated three times for each ecDNA to test the GMM, and each indicator was averaged over three repeats.

### ecDNA and eccDNA identification

The processed WGS FASTQ files were aligned to the GRCh38 using BWA-MEM^58^. Copy number variations (CNVs) were called from CNVkit^59^. The seed intervals were generated from CNVs with a default threshold of copy number ≥ 4.5 and interval size ≥ 50 kb. AmpliconArchitect (AA) was applied to detect focal amplifications and reconstruct the amplicon structures using an indexed BAM file and seed intervals as input^60,61^. Amplicons were subsequently categorized as ecDNA amplicons, breakage-fusion-bridge (BFB), and complex non-cyclic or linear amplicons by AmpliconClassifier (AC)^62^. Experimentally verified ecDNA breakpoints were converted to hg38 using the R package liftOver (v.1.24.0)^7^.

We treat scATAC-seq as a pseudo-bulk ATAC-seq library for ecDNA identification, employing a strategy similar to our previously published tool^10^, with optimizations tailored for single-cell data. The bam file from a scATAC-seq is significantly larger (~50 GB) compared to a bulk ATAC-seq (~10GB). Therefore, we integrated the multiprocess model (v.0.70.14) for parallel processing, where each process handles a given window size of the genome (default window size = 64,000,000). Additionally, we optimized functions using Cython (v.0.29.33), which improved execution speed and made the framework more efficient compared to our previous work. The method for assembling ecDNA from breakpoint graphs is carried over from our previously published tool^10^. The entire ecDNA identification process is executed within Python (v.3.10.12) environment, and all parameters can be viewed using the circlehunter2-h command. After identifying ecDNA, we retained ecDNAs with sizes ranging from 10 kb to 20 Mb, depth_mean > 5, and high_coverage > 0.1 to exclude false positives.

For Circle-seq and bulk ATAC-seq data, quality control was performed using fastp^63^ and alignment to the hg38 reference genome was performed using BWA-MEM^58^. Circular DNA was identified using Circle-Map and ATACamp^21,22^. For Circle-Map results, we applied the following filters to reduce false positives: score > 200, split reads between 5 and 10, coverage increase at the start coordinate ≥ 0.33, and coverage increase at the end coordinate ≤ 0.33.

### Assigning ecDNA to cells

The process of distinguishing cell populations harboring ecDNA is carried out within the R environment. First, we calculate the normalized ATAC signals for cells within the ecDNA region based on the fragment.tsv output by cellranger-atac. Read counts within the ecDNA region are normalized using counts per million (CPM) per cell

$$\log 2({{ecCPM}}_{i})=\log 2\left(\frac{{reads\; within\; ecDNA\; for}{{cell}}_{i}}{{all\; mapped\; reads\; for}{{cell}}_{i}}\times 1{e}^{6}\right)$$

where ecCPM_*i*_ is the CPM for the *i*th cell within the ecDNA.

Next, a non-unimodal test is performed using the dip.test function from the diptest package (v.0.77.1). If the *P*-value < 0.05, we infer that the normalized signal distribution of all cells follows a mixed model and fit a two-component normal distribution (*k* = 2) using the normalmixEM function from the mixtools package (v.2.0.0) according to the formula^18,64^

$${lo}g2\left({ec}{CPM}\right) \sim w{\mathscr{\times }}{\mathscr{N}}\left({\mu }_{1},{\sigma }_{1}^{2}\right)+(1-w{\mathscr{)}}{\mathscr{\times }}{\mathscr{N}}\left({\mu }_{2},{\sigma }_{2}^{2}\right)$$

where *μ*₁ and *μ*₂ denote the means of the two Gaussian models, *σ₁* and *σ₂* represent the estimated standard deviations of the respective models, and *w* is the estimated weight parameter of the first Gaussian model.

If the *P*-value > 0.05, we apply *k*-mean clustering (*k* = 2) to the GeneScore matrix of genes located on the ecDNA. We utilize the GeneScore matrix instead of PeakMatrix to mitigate the sparsity issues inherent in scATAC-seq data. Subsequently, we use samtools (v.1.11) to extract cells with discordant reads at the ecDNA breakpoints from the BAM file, which are treated as high-confidence cells. Finally, a Fisher’s exact test is conducted using the fisher.test function from the janitor package (v.2.2.0) to assess whether discordant reads were enriched in cells inferred to carry ecDNA. scCirclehunter is available for use online at https://github.com/suda-huanglab/scCirclehunter.

### Extract reads from pooled scATAC-seq library

PISA (v.1.0) was used to parse out the cell barcodes and UMIs based on the raw FASTQ files of scATAC-seq data^65^. Clean reads were aligned to the hg38 reference genome by BWA-MEM^58^. dnbc4tools (v.2.1.3) was used to generate fragment.tsv from bam file. The fragment.tsv file from the local scATAC-seq run was imported and analyzed using the ArchR package. Processed scRNA-seq data with annotated cell lines were downloaded and imported into Seurat^20^. For each pooled scATAC-seq library containing multiple cell lines, the corresponding scRNA-seq data for the same cell line were integrated, and the addGeneIntegrationMatrix function from the ArchR package was used to assign cell identities from the scRNA-seq data to the cells in the scATAC-seq library. Next, the subset-bam_linux (v.1.1.0) tool was employed to extract the reads of cells annotated as SW480 from the BAM file. The SW480 cell-derived reads from two samples were then merged, yielding a total of 1297 SW480 cells, which aligns with the 1269 SW480 cells reported in the published research. A similar approach was applied to isolate the DLD1 cell-derived reads.

### Creating mixtures of ecDNA^+^ and ecDNA^‒^ cells

For the 1297 SW480 (ecDNA^+^) and 1076 DLD1 (ecDNA^‒^) cells, the proportion of ecDNA^+^ cells varied from 10% to 90% in 10% increments. A total of 1000 cells were randomly sampled from the two colon cell lines according to the specified proportions. Our model was then applied to classify the two cell lines based on the identified ecDNA region. Each proportion was repeated 10 times, and the model’s performance was averaged across multiple replicates.

### Metacells construction

We implemented a metacell-based approach for single-cell correlation analysis. Specifically, we initialized Seurat object using the SetupForWGCNA function from the hdWGCNA package (v.0.4.5)^66^. Subsequently, malignant cells were grouped by patient origin and aggregated into metacells via the MetacellsByGroups function. Finally, metacells were extracted using the GetMetacellObject function and stored as Seurat object. Key parameters included: *k* = 10 nearest neighbors for cell merging and max_shared=5 for maximum overlap. Finally, gene expression correlations were calculated using Pearson’s correlation coefficient on the resultant metacells.

### CIBERSORTx estimation

CIBERSORTx was used to determine the proportion of each cell type in TCGA-GBM samples^67^. The reference IDH-wt GBM single-cell dataset was GSE131928, and cell type annotation was based on previous article^14^. We annotated 7930 cells, including OPC (*n* = 1753), NPC (*n* = 893), AC (*n* = 3431), MES (*n* = 556), macrophages (*n* = 978), oligodendrocytes (*n* = 223), and T cells (*n* = 96). For each cell type, a random subset of 200 cells was selected as the training data for CIBERSORTx. Subsequently, mRNA Affymetrix data from the 47 TCGA-GBM samples with ecDNA information were uploaded to estimate the cell composition of the samples. For amplified genes associated with cellular states in GBM, including *EGFR*, *PDGFRA*, and *CDK4*, the 47 GBM patients were classified into three groups: ecDNA, ChrAmp, and NoAmp. The amplification state of these genes was obtained from the TCGA copy number data.

### GSEA

GSEA analysis of the TCGA expression data was performed using the gggsea package (v.0.1.0). The target genes of FOXG1 were obtained from a published article, and a threshold of adj.*P* < 0.05 and FoldChange > 1 was applied to filter the list of FOXG1 target genes, resulting in 84 genes retained for further analysis (Supplementary Table S5)^68^. mRNA affymetrix data from 47 TCGA-GBM samples with ecDNA identification information were categorized into two groups: ecGSX2 and non-ecDNA groups. We used the limma package (v.3.56.2) to assess fold change.

To identify differentially expressed genes in ecNR2E1^+^ malignant cells, FindAllMarkers function from Seurat package was used and genes were filtered based on a *P*-value < 0.05 and |avg_log_2_FC| > 0.5, resulting in 1045 highly expressed genes. Pathway enrichment analysis was performed using the enrichGO function from the clusterProfiler package (v.4.8.1)^69^, with a focus on pathways significantly enriched (*P*-value < 0.01) and containing the *NR2E1* gene.

For scRNA-seq enrichment analysis, the scGSVA package (v.0.0.22) was used to score the activity of all Gene Ontology (GO) terms (*n* = 22,964) in the scRNA-seq data, with the ssgsea scoring method selected. Differential test between discordant+/‒, receiver/non-receiver groups was assessed using the wilcox.test function, and adjusted *P*-values were calculated using the p.adjust function with the Benjamini–Hochberg method.

### Mitochondrial transfer analysis

The scRNA-seq data of GBM and RCC were analyzed using the MERCI framework to infer mitochondrial transferring^41^. mtSNVs were called using MERCI-mtSNP with default parameters. Subsequently, identity inference was performed using the MERCI R package. The mutation file was read using readMTvar_10x with the minReads parameter set to 10, and the coverage file was read using readCoverage_10x. Non-malignant cells were considered as donor cells, while malignant cells were assumed to be the recipients of the hijacked mitochondria. The MTmutMatrix_transform function was used to generate the corresponding vaf matrix for mtSNVs with min_*d* = 2 and min_observeRate = 0.05. The functions Denrich_mtMut_extr, Cell_Neff_cal, and MERCI_LOO_MT_est were applied to estimate the DNA rank and RNA rank, using default parameters. The CellNumber_test function was used to calculate the RCM values in 10% increments within the range of 10% to 100%. RCM > 1 (empirical *P*-value < 0.0001) was considered significant. The percent with the highest RCM value was used as the input for the top_rank parameter in the MERCI_ReceiverPre function.

### RCC malignant cell annotation

To ensure reproducibility, we applied Cancer-Finder to identify malignant cells within RCC scRNA-seq data (nSample = 19)^44^. This deep learning-based tool requires only a Cell*Gene matrix as input. Pre-trained Cancer-Finder models (sc_pretrain_article.pkl and model_epoch92.pkl) were obtained from the GitHub repository (https://github.com/Patchouli-M/SequencingCancerFinder). Malignant cell annotations were generated using default parameters, where “0” denotes normal cells and “1” indicates malignant cells.

### Hi-C

BAM files for hic data were downloaded directly from EGA, then converted to .pairs format using bam2pairs. Following this, Juicebox was employed to convert the .pairs files into .hic format^70^, and HiCLift (v.1.0) was applied to lift the .hic files to the hg38 version^71^. For downstream analysis, the HiCExperiment (v.1.0.0) and HiContacts (v.1.5.0) packages were used^72^. Contact matrix involving ecDNA regions were plotted at 5 kb resolution, while inter-chromosomal connections involving ecDNA were visualized at 50 kb resolution using the circlize R package (v.0.4.15)^73^.

Hi-C data normalization was performed using Iterative Correction and Eigenvector decomposition (ICE), followed by chromatin loop calling with the pyHiCCUPS algorithm at 10 kb resolution and with the maximum genomic distance set to 5 Mb^74^. Loop anchors were annotated to corresponding genes using the ChIPseeker package (v.1.36.0)^75^.

We implemented a published computational framework to correct for the effects of copy number on chromatin contact frequency^24^. This method partitions the genome into 500 kb bins and quantifies: (i) observed interaction frequency: proportion of bin-ecDNA contacts relative to all contacts on the same chromosome. (ii) expected interaction frequency: bin copy number relative to total copy number on the same chromosome. Under the null hypothesis that bin-ecDNA interaction is weighted only by its underlying copy number, a binomial distribution model is used to compute the statistical significance of observed-versus-expected contacts. *P*-values were Bonferroni-corrected for genome-wide multiple testing to identify significant interactions (FDR < 0.05), and bins with adjusted *P* value < 0.05 were selected as significant interacting regions.

### TF regulatory network construction

To identify candidate TF target genes, we used paired scATAC-seq and scRNA-seq data and followed the previously published TF network construction method^36^. For each TF, we defined candidate target genes using two criteria: (1) the target gene’s promoter region directly contains the TF’s binding motif, and (2) the target gene’s promoter region is linked through gene-associated candidate cis-regulatory elements (cCREs). DNA sequence motifs within peaks were added with the AddMotifs function from the Signac package, using the “human_pwms_v2” position frequency matrix from the cisBP database.

Candidate cCREs were identified using the Cicero R package (v.1.18.0). We computed Pearson correlations as co-accessibility scores for each peak-to-peak link with the run_cicero function (co-accessibility cutoff of 0.2). Next, we selected links where one peak overlapped a gene promoter region (within 1 kb of the transcription start site) and then calculated the Pearson correlation between the averaged chromatin accessibility of the second peak and the gene’s averaged RNA expression across all clusters. Significant gene-linked cCREs were identified by applying a Benjamini-Hochberg corrected *P*-value threshold of < 0.05, yielding 2908 high-confidence candidate cCREs. Finally, the *NR2E1* regulatory network was constructed from these TF–gene pairs using the igraph R package (v.1.5.1). Code is available at https://github.com/Dragonlongzhilin/RenalTumor.

### Unsupervised trajectory inference using monocle3

To prepare inputs for monocle3 (v1.3.1) using Signac (v1.8.0)^76,77^, we first created a ChromatinAssay object from a count matrix with the CreateChromatinAssay function, followed by generating a Seurat object with CreateSeuratObject. Frequency-inverse document frequency (TF-IDF) normalization and dimensional reduction were applied using RunTFIDF, FindTopFeatures, and RunSVD. UMAP coordinates from ArchR were imported into the Seurat object. The Seurat object was then converted to a cell_data_set object using the as.cell_data_set function from the SeuratWrappers package (v0.3.1). Clustering was performed using cluster_cells function of monocle3, and a principal graph was learned from the reduced dimensional space via the learn_graph function, with learn_graph_control set to ncenter = 70. The order_cells function designates the ecMDM4_neg OPC cells as the root state.

### Estimating ecDNA amplicon copy number

Following the established method, we estimated the copy number for ecDNAs based on background ATAC-seq signals^5,78^. Specifically, we applied a sliding window of 5 Mb with 1-Mb increments across the hg38 reference genome. For ecDNA copy number estimation, z-scores were calculated for the following genomic intervals: chr7:51,000,001–56,000,000 for EGFR-bearing ecDNAs, chr4:52,000,001–57,000,000 for ecPDGFRA, chr1:203,000,001–208,000,000 for ecMDM4, and chr12:64,000,001–69,000,000 for ecMDM2. For MYC-bearing amplicons in COLO320DM and COLO320HSR, the interval chr8:124,000,001–129,000,000 was selected^5^.

The code is available at https://github.com/GreenleafLab/10x-scATAC-2019.

### Survival analysis

Survival analysis were conducted with Kaplan–Meier method using R packages survival (v.3.6.4) and survminer (v.0.4.9). For the 482 GBM patients with available survival information, patients were classified into two groups based on normalized mRNA Affymetrix microarray expression data: those with *NR2E1* expression in the top 50% were assigned to the *NR2E1*-high group, while the remaining patients were assigned to the *NR2E1*-low group. Overall survival analysis is performed by the Log-rank test, and *P* < 0.05 is considered significant.

### GBM cell type and cellular state annotation

The marker genes for each cell type were sourced from published articles. The cell annotations (including malignant cells) for the two large datasets, GSE174554 and EGAD00001010313, were downloaded from GSE174554 and GSE226726, respectively, and the cell composition for other single-cell datasets was consistent with the descriptions provided in the original articles from which the data were derived. The markers used for other cell types were as follows: macrophages (*CD14, AIF1, FCER1G, FCGR3A, TYROBP, CSF1R*); T cells (*CD2*, *CD3D*, *CD3E*, *CD3G*); and oligodendrocytes (*MBP*, *PLP1*, *MAG*, *MOG*, *CLDN11*). We downloaded the marker genes for four distinct GBM cellular states from published studies^14,31^. For both scRNA-seq and scATAC-seq data, we used the addModuleScore function to assign each GBM cell to the cell state with the highest score. Given that the marker genes were derived from transcriptomic data, and cellular states within scATAC-seq clusters were not homogeneous, we employed nonnegative matrix factorization (NMF) to refine the score-based annotations. Specifically, we used the nmf function from the NMF R package (v.0.21.0) with the rank parameter set to 4, which identified four distinct groups. We observed that each of the four GBM states was predominantly represented in a single group (with more than 50% of cells in each group corresponding to a particular GBM state). As a result, each group was assigned a cell state, and the final NMF-corrected annotation was consistent within the cell clusters.

### Motif enrichment analysis

Differentially accessible peaks within ecDNA regions were identified using the getMarkerFeatures function to compare cells in the ecDNA^+^ and ecDNA^‒^ groups. Differentially accessible elements in the ecDNA^+^ cluster were filtered based on an FDR ≤ 0.05 and a log_2_FC ≥ 1. A total of 327 peaks within the ecPDGFRA region were significantly upregulated in the ecDNA^+^ cluster. Motif discovery and analysis were performed using HOMER (v.4.11). Log2_Enrichment was calculated as log2(percentage of target sequences with motif/percentage of background sequences with motif). A log_2__Enrichment > 0.5 and a *P*-value < 1e‒20 were considered statistically significant.

### Statistical analysis

Details of all statistical tests used can be found in the corresponding figure legends. If not otherwise specified, statistical significance was assessed using the two-sided Wilcoxon test. ns, not significant; *P < 0.05; **P < 0.01; ***P < 0.001; ****P < 0.0001.

### Data visualization

The circular structure of ecDNA was visualized using the circlize package (v.0.4.15), and the gTrack package (v.0.1.0) was employed to plot the segment links and depth of ecDNA. Additional visualization tools included ggplot2 (v.3.5.1), ggpubr (v.0.6.0), ggcoverage (v.1.4.0), pheatmap (1.0.12), ComplexHeatmap (v.2.15.4), et al. All visualizations were performed in the R environment (v.4.3.2).

## Supplementary information

Supplementary Information

Supplementary Table S1

Supplementary Table S2

Supplementary Table S3

Supplementary Table S4

Supplementary Table S5

Supplementary Table S6

Supplementary Table S7

Table S8

## Data Availability

The research is based on public data. The raw WGS data for the SW480 and DLD1 cell lines is from the SRA database with the accession numbers SRR8670707 and ERR2817158. The raw and processed scRNA-seq and scATAC-seq data involving SW480 and DLD1 are available in CNGBdb with accession numbers CNP0004330 and CNP0003658. The raw and processed scATAC-seq data for GBM are obtained from GSE139136^79^ and EGAD00001010313/GSE226726^28^. Processed GBM scRNA-seq data are obtained from GSE174554^29^ and GSE131928^14^. Paired GBM scATAC + scRNA-seq raw data are retrieved from GSE230389^80^. The 10X scATAC-seq data of SW480 is from GSE196515. The scATAC-seq data of COLO320DM and COLO320HSR are from GSE160148^5^. Hi-C sequencing data are obtained from EGAD00001010312^28^. The TCGA-GBM data are publicly available. Processed ATAC-seq, RNA-seq, and copy number data for GBM samples from different spatial regions are downloaded from the supplementary materials of previously published research^28^. The raw and processed scRNA-seq and scATAC-seq data for ccRCC are obtained from GSE207493^43^. The details for other available scATAC-seq datasets are provided in the supplementary table. We reference ecDNA structures of cell lines collected in published databases^81,82^. Main datasets in the manuscript are summarized in the Supplementary Table S8.
