# Supplementary material for: scCirclehunter delineates ecDNA-containing cells using single-cell ATAC-seq, with a focus on glioblastoma
Source: Cell Discov. 2025 Dec 9;11:98. doi: 10.1038/s41421-025-00842-9 (PMC12686523; doi:10.1038/s41421-025-00842-9)
Supplement: Supplementary file 1 — Supplementary Information [file 41421_2025_842_MOESM1_ESM.pdf]

Supplementary Figures

Supplementary Figure 1.

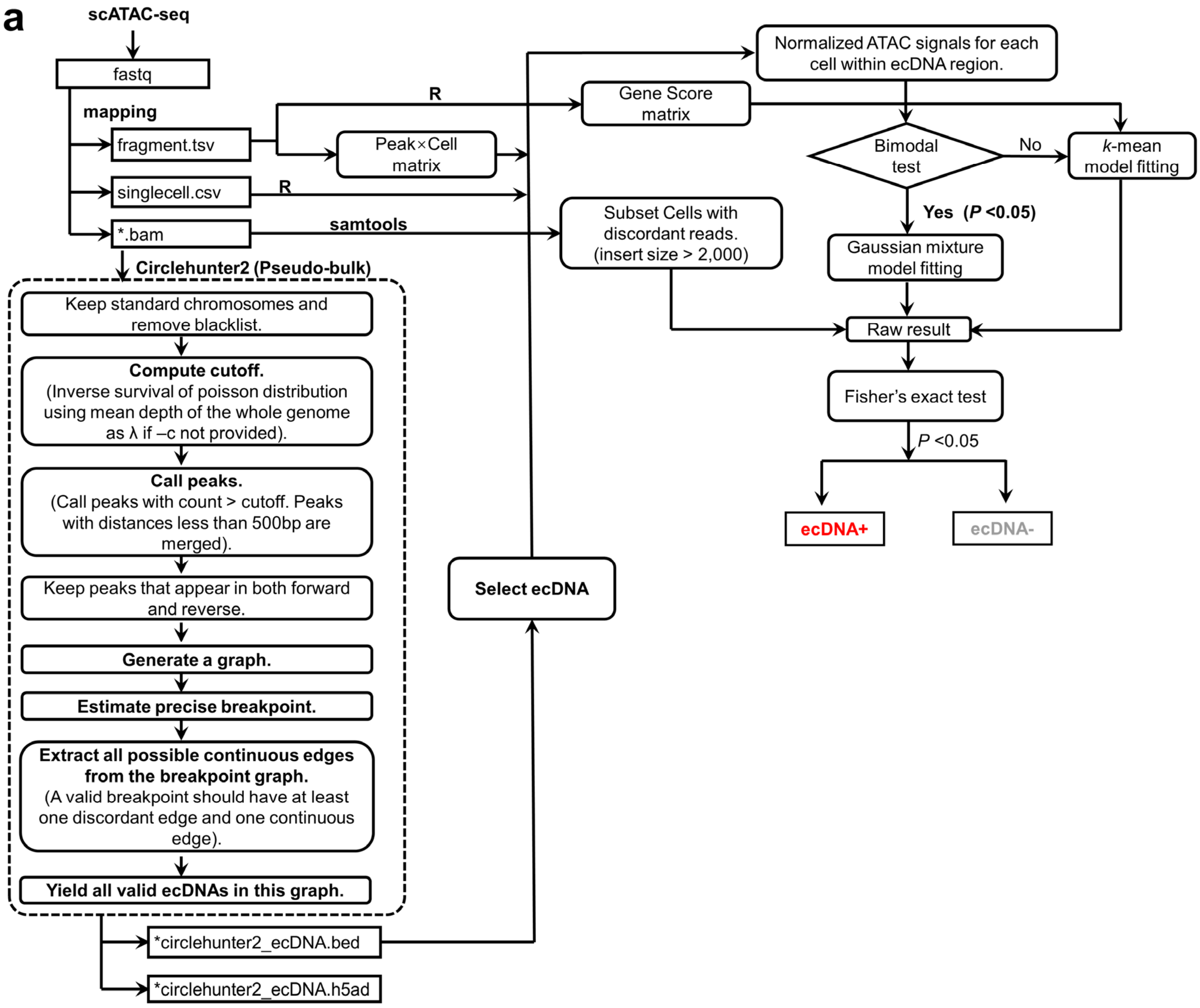

Supplementary Fig. S1.

Flowchart of scCirclehunter algorithm.

a Workflow of scCirclehunter used to predict candidate ecDNA along with cells containing the ecDNA.

Supplementary Figure 2.

a

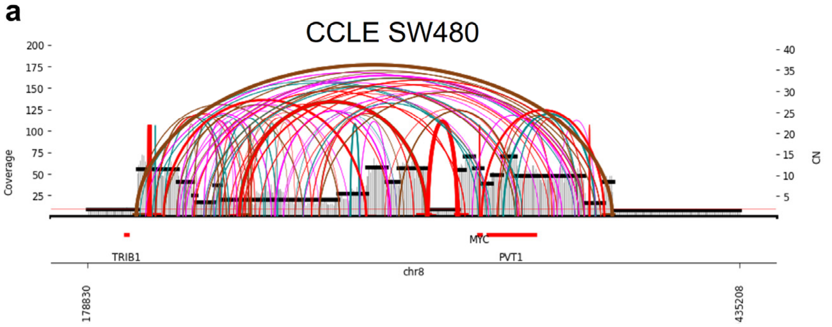

b

Chrom	Start	End	Copy number
chr8	125498829	126063824	8.33
chr8	126893815	128608799	8.79

c **d** **e**

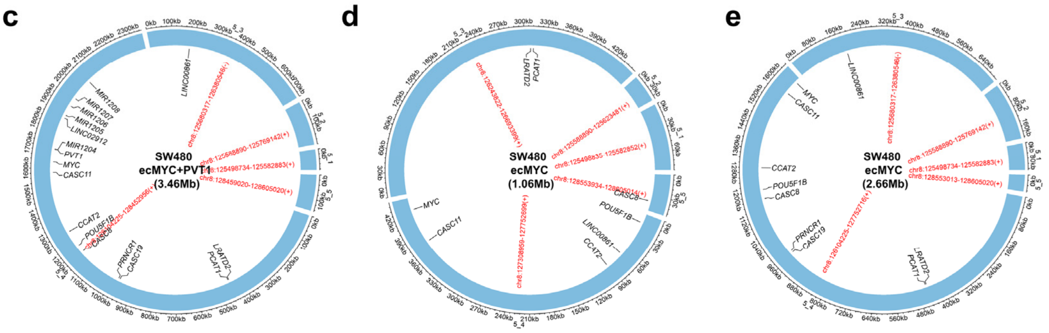

f

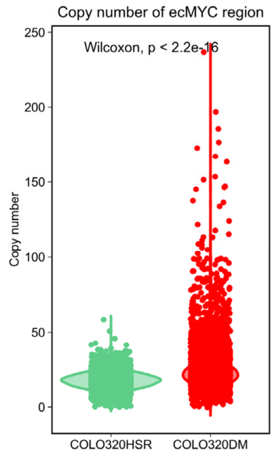

g **h**

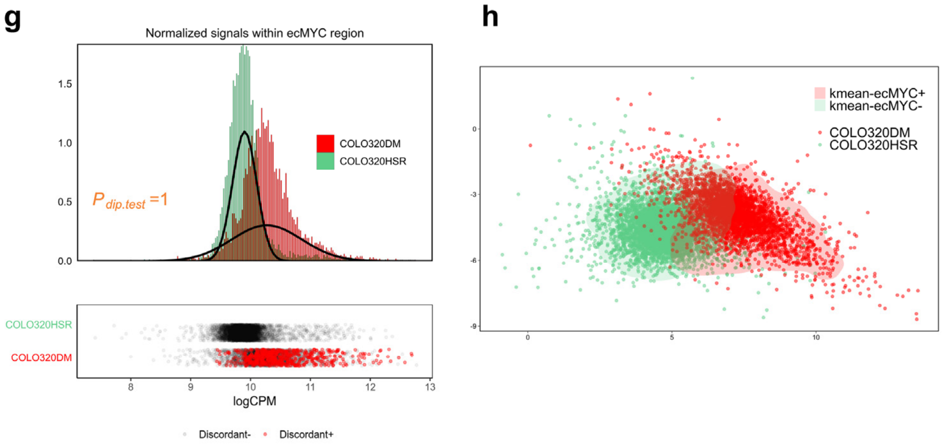

i

Method	Precision	Recall	F1-score
GMM	0.758	0.505	0.606
k-mean	0.836	0.762	0.797

Supplementary Fig. S2.
Performance of scCirclehunter on real data.
a ecMYC amplicon structure in the SW480 cell line identified by AA. **b** The seed intervals with high copies detected in the SW480 cell line by CNVkit. **c, d, e** ecMYC amplicons 1 **(c)** and 2 **(e)** detected in the 10X scATAC-seq library of SW480, and ecMYC identified in the pooled scATAC library **(d)**. **f** The copy number of ecMYC region for COLO320DM and COLO320HSR cells based on scATAC-seq data. **g** Apply Gaussian decomposition to classify ecMYC+ (COLO320DM) and ecMYC- (COLO320HSR) cells, with COLO320DM cells carrying discordant reads marked in red. **h** K-means clustering (n=2) was used to cluster the Gene Score of genes on ecMYC to classify ecMYC+ (COLO320DM) and ecMYC- (COLO320HSR) cells. **i** The performance of Gaussian decomposition and k-means clustering in predicting COLO320DM and COLO320HSR cells based on the known ecMYC region.

Supplementary Figure 3.

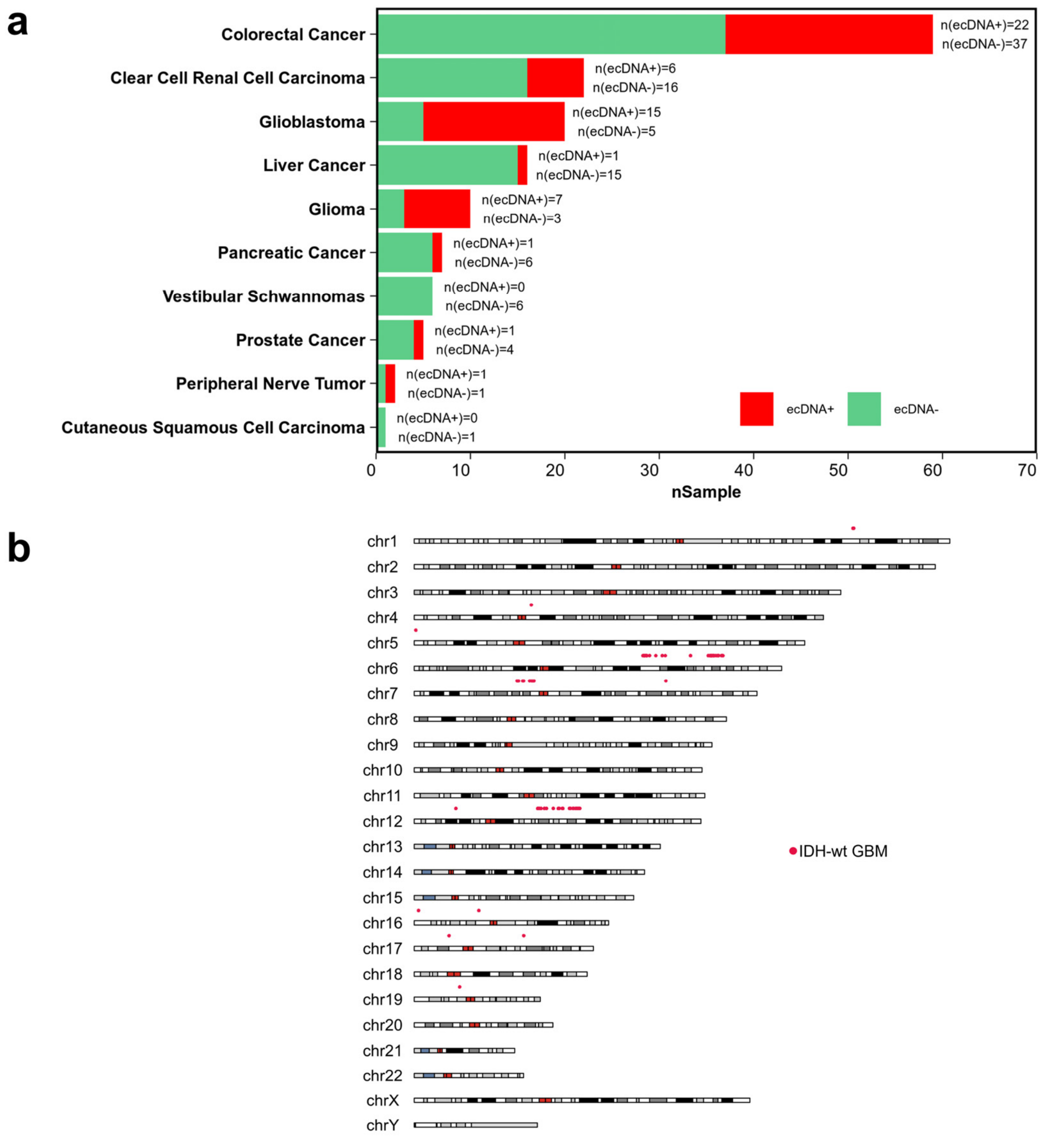

Supplementary Fig. S3.

Detection of ecDNA based on available scATAC-seq data.

a Bar plot showing the detection of ecDNA from scATAC-seq data across 148 samples from 10 tumor types. **b** Karyotype plot showing the chromosomal distribution of ecDNAs identified in 20 IDH-wt GBM samples from 13 patients.

Supplementary Figure 4.

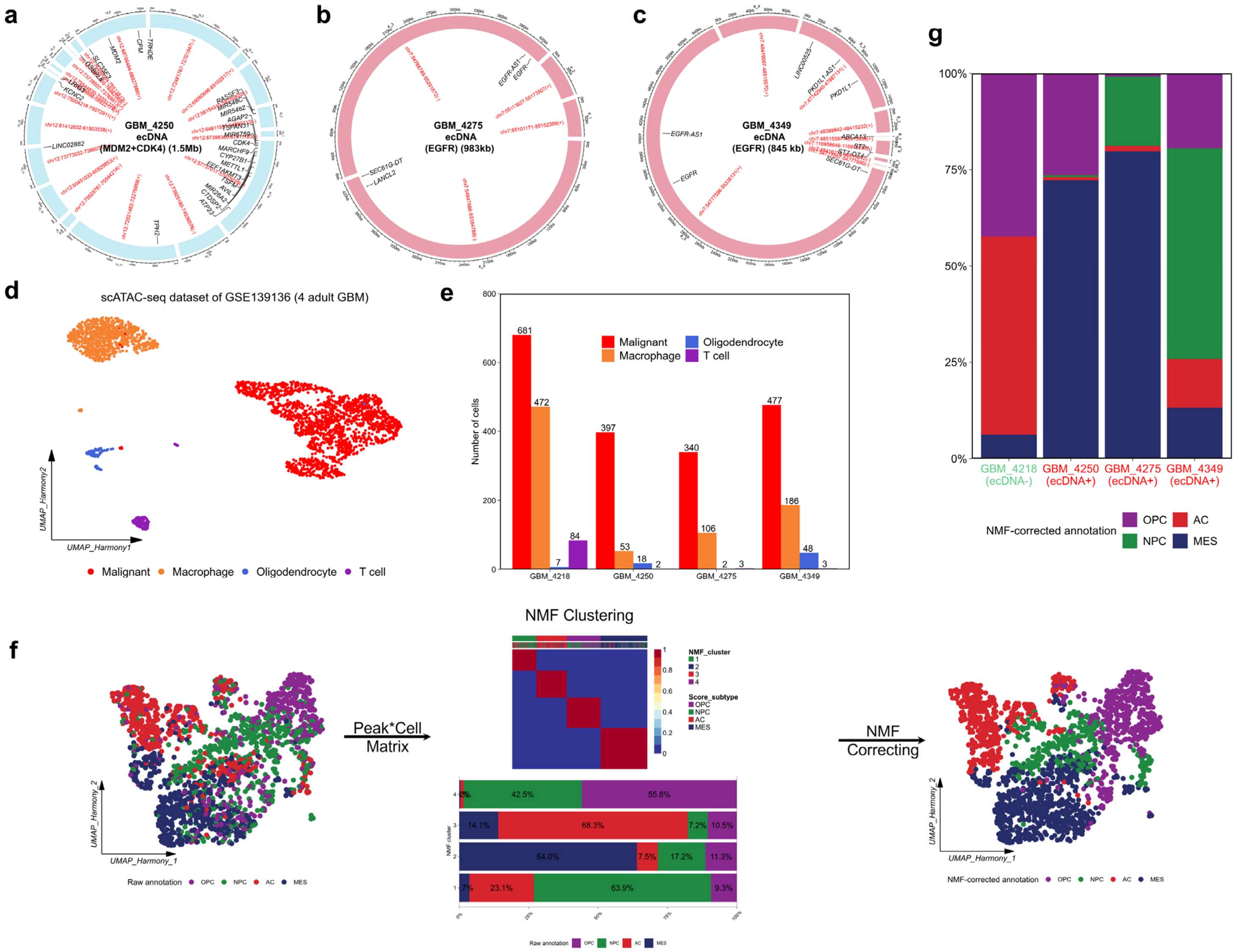

Supplementary Fig. S4.

Construction of ecDNA structures and cell annotations based on scATAC-seq data from four adult GBM patients.

a,b,c Construction of ecDNA structures, including ecMDM2 from GBM4250 **(a)** ecEGFR from GBM4275 **(b)**, and ecEGFR from GBM4349 **(c)**. **d, e** Cell type annotations **(d)** and composition **(e)** based on scATAC-seq data from four adult GBM patients, including malignant cells, oligodendrocytes, macrophages, and T cells. **f** Cellular state assignments for malignant cells from four patients based on meta-module gene list scoring, with classification correction performed using NMF. **g** Cellular state composition of malignant cells in the four GBM patients.

Supplementary Figure 5.

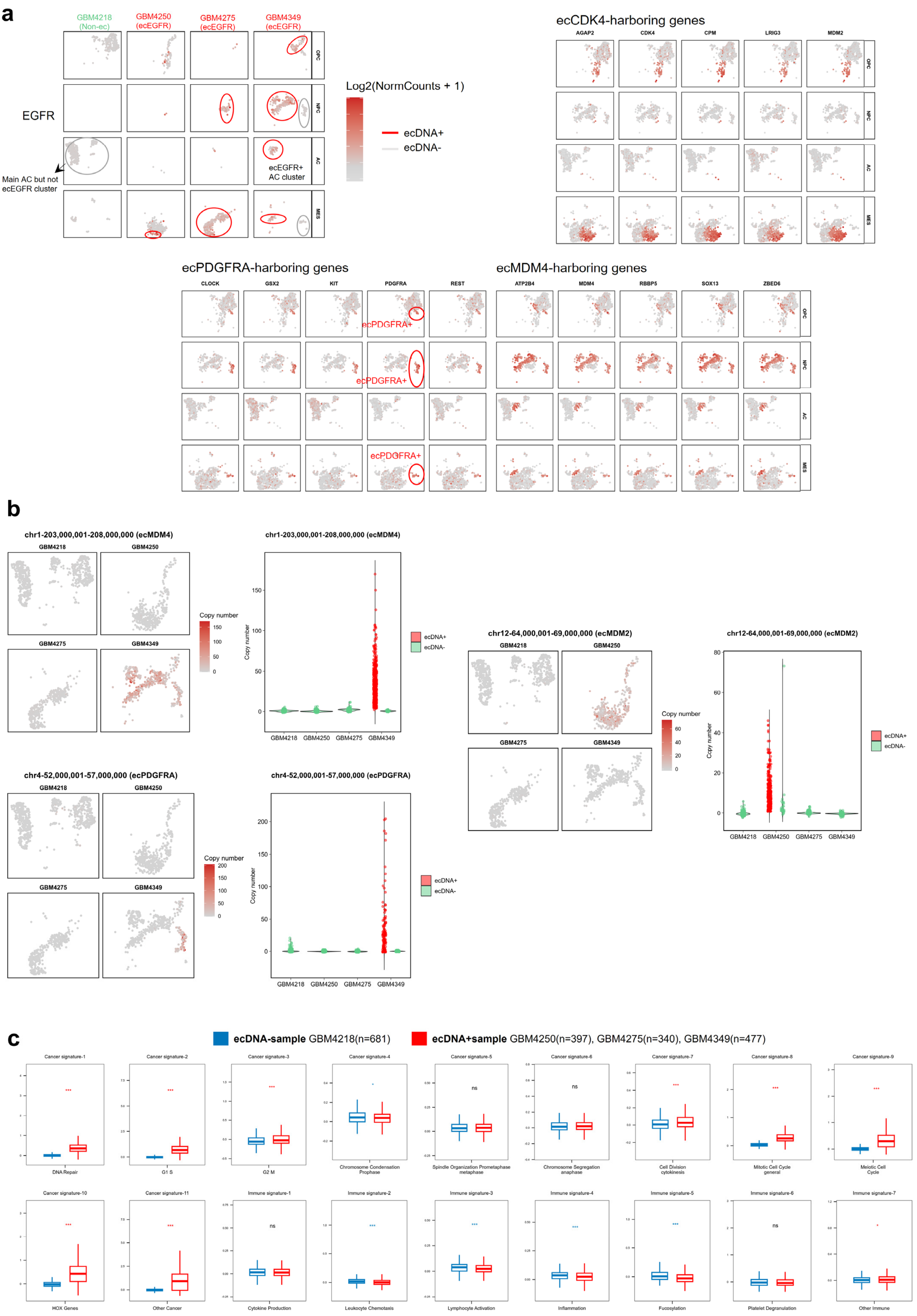

Supplementary Fig. S5.

Heterogeneity in accessible chromatin signals and gene copy numbers carried by ecDNA across GBM patients.

a The normalized scATAC-seq signal of genes within the amplified regions of ecDNA derived from malignant cells of four GBM patients. **b** Estimated copy number z-scores for ecDNA regions. **c** Scoring of 18 ecDNA-related gene sets in malignant cells, including 11 upregulated and 7 downregulated signatures. Malignant cells were classified based on their patient origin: one patient (GBM4218) did not have ecDNA, while three patients (GBM4250, GBM4275, and GBM4349) had ecDNA.

Supplementary Figure 6.

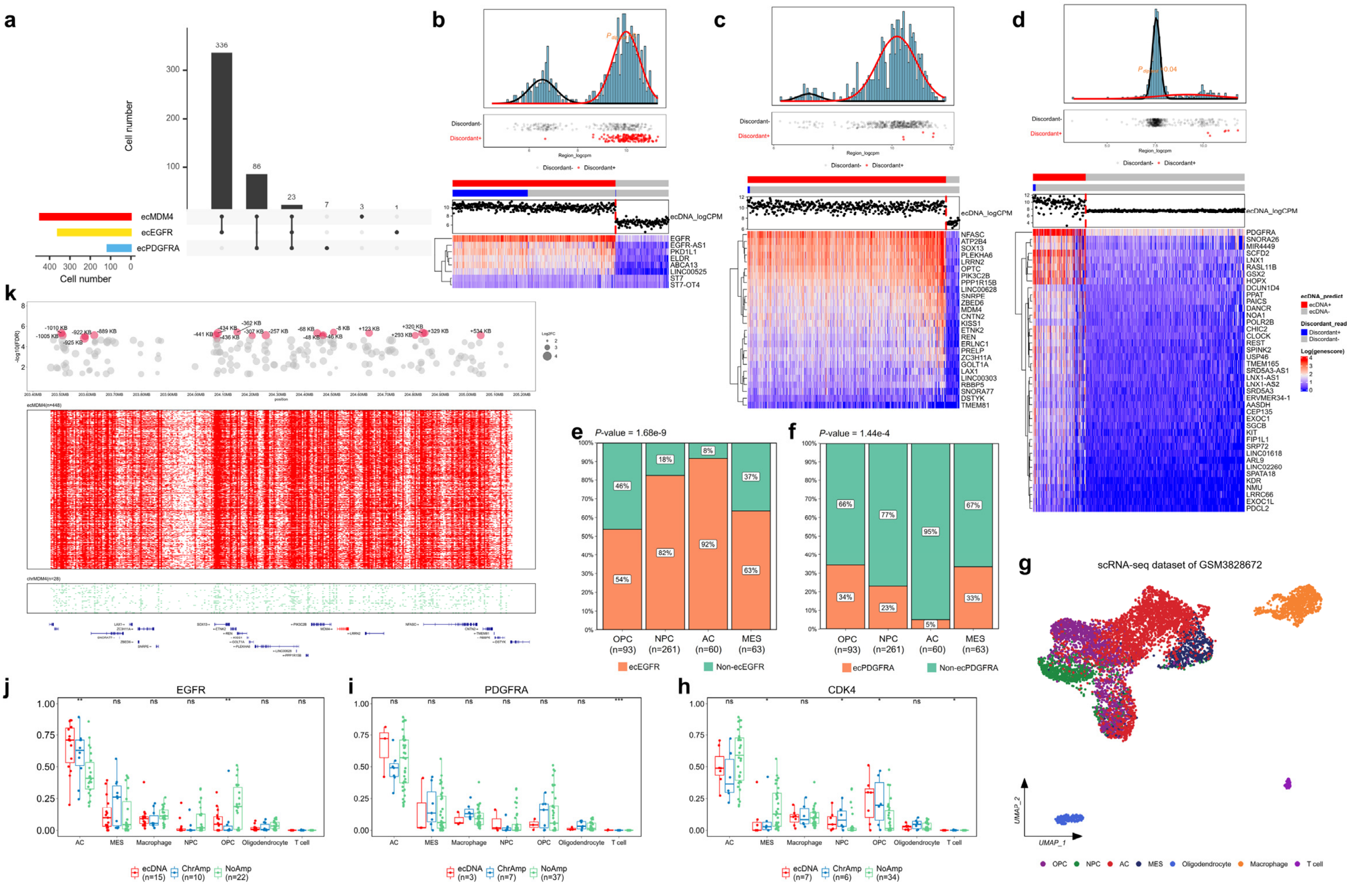

Supplementary Fig. S6.

Association between ecDNA amplification and malignant cellular states.

a Upset plot displaying the co-occurrence of the three ecDNAs identified in GBM4349 (*MDM4*, *EGFR*, *PDGFRA*) in single cells (n = 477). **b, c, d** Gaussian mixture model for predicting whether cells carry ecEGFR, ecMDM4, and ecPDGFRA. **e, f** Chi-squared test assessing significant differences in ecEGFR (E) / ecPDGFRA (F) distribution between cellular states in the GBM4349 patient, with a *P*-value < 0.05 considered statistically significant. **g** scRNA-seq data dimensionality reduction and cell annotation results for the GSM3828672 dataset, used as a reference for CIBERSORTx. **h, I, j** Cellular composition of 47 TCGA-GBM patients with ecDNA information was estimated from mRNA Affymetrix data using CIBERSORTx. The patients were divided into three groups: ecDNA, ChrAmp, and NoAmp, based on the presence of ecDNA carrying *CDK4* **(h)** *PDGFRA* **(i)**, and *EGFR* **(j)**. Statistical significance was assessed using the Kruskal-Wallis test, with a *P*-value < 0.05 considered significant. **k** Malignant cells carrying ecDNA versus those without. Top to bottom, differentially accessible peaks within the ecDNA region for cells carrying ecDNA compared to those without. The top 20 peaks with the highest log2FC were identified, and the distances to the *MDM4* promoter were calculated and annotated. Bigwig signals for each ecDNA+ cell within the ecDNA region. Bigwig signals for each ecDNA- cell within the ecDNA region. Gene annotations are provided.

Supplementary Figure 7.

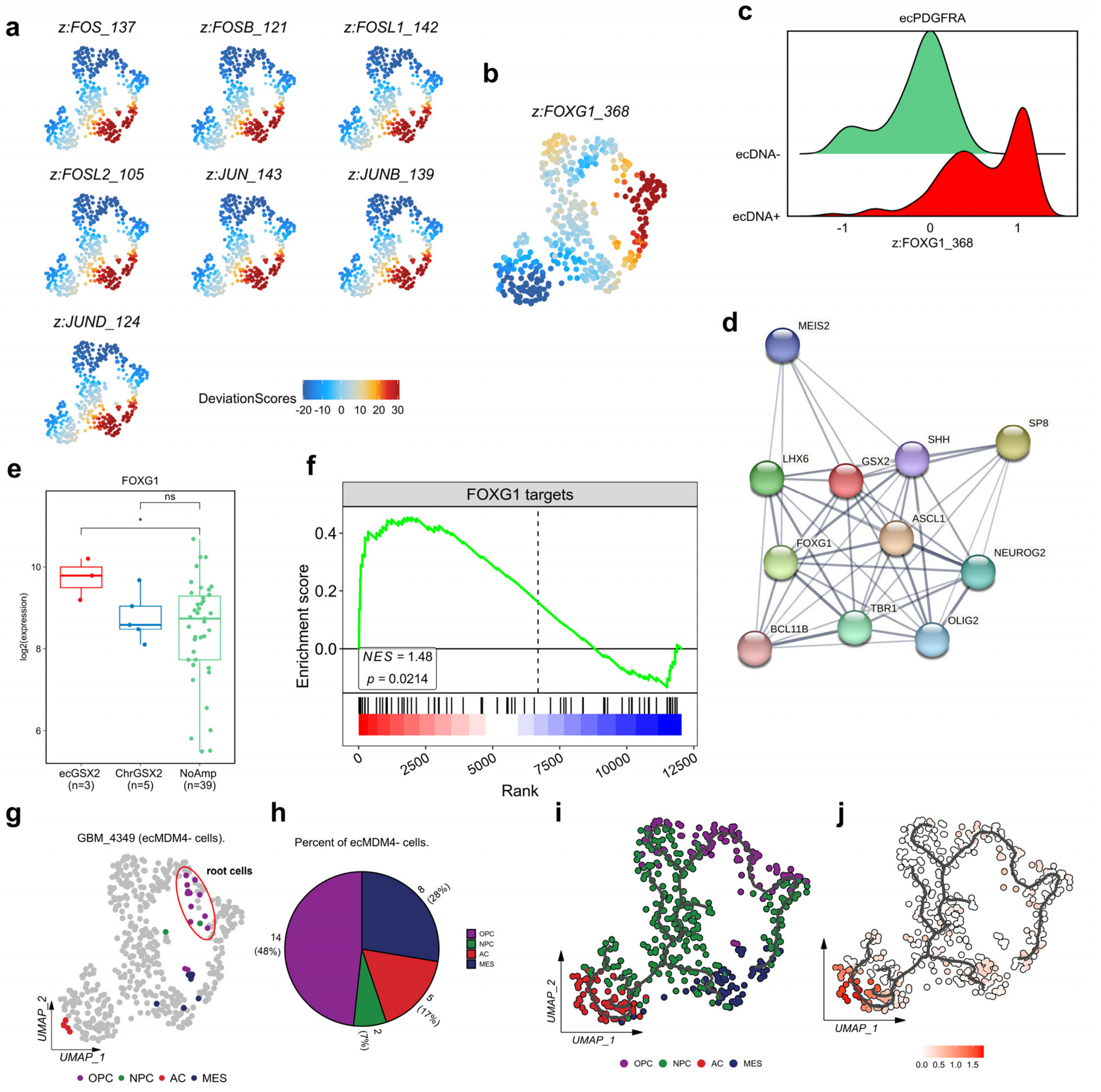

Supplementary Fig. S7.

Analysis of downstream genes regulated by ecDNA and potential trajectories in the GBM4349 patient. **a** Transcription factor activity of the *AP-1* family in GBM4349-derived malignant cells. **b** Transcription factor activity of *FOXG1* in GBM4349-derived malignant cells. **c** Transcription factor activity of *FOXG1* in GBM4349-derived malignant cells, grouped by ecPDGFRA state. **d** *GSX2* regulated *FOXG1*, as identified through STRING network analysis. **e** Differential expression of *FOXG1* according to the groups of ecGSX2 (n = 3) chromosomal *GSX2* amplification (n = 5) and non-GSX2 amplification (n = 39). Statistical significance was assessed using a two-sided Wilcoxon test. **f** GSEA plot of genes involved in *FOXG1* targets, grouped by ecGSX2 (n = 3) and non-ecGSX2 (n = 44). **g** UMAP plot showing cells that did not carry ecMDM4. **h** Pie plot showing the composition of cellular states for ecMDM4- cells. **i, j** Trajectories of malignant cells in GBM4349, inferred using monocle3, annotated by cellular states **(i)** and pseudo-time **(j)**.

Supplementary Figure 8.

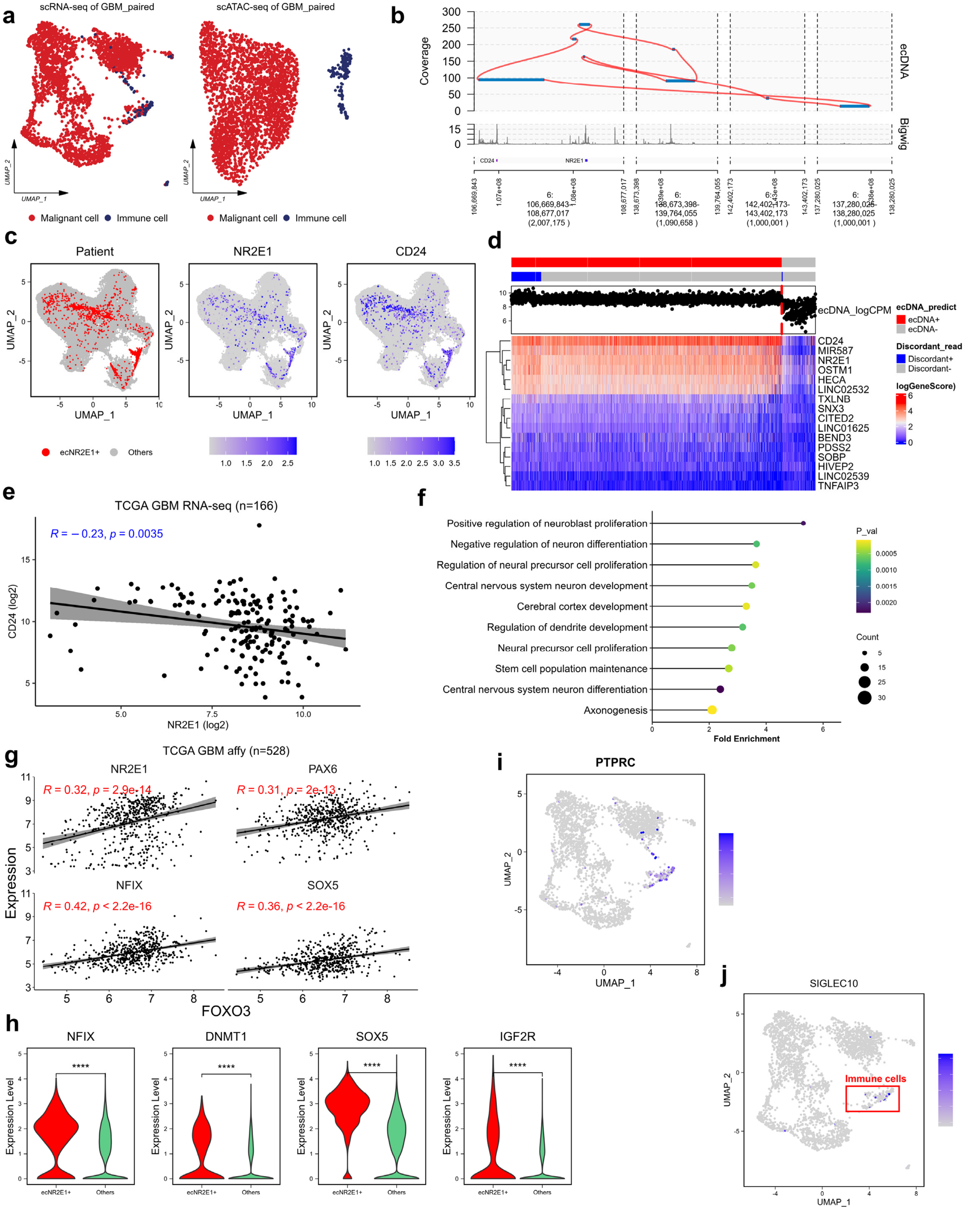

Supplementary Fig. S8.

Analysis of cell annotation and ecDNA-induced heterogeneity in GBM patients.

a Cell type annotation of paired scRNA-seq and ATAC-seq data from a GBM patient, including malignant cells (n = 2,739) and immune cells (n = 150). **b** Sequencing depth and linkage of the chromosomal segments constituting ecNR2E1. **c** Integration and dimensionality reduction of malignant cells (n = 2,739) from scRNA-

seq data of the ecNR2E1 patient with malignant cells (n = 80,482) from snRNA data of other GBM patients revealed that *NR2E1* and *CD24* were specifically expressed in malignant cells derived from the ecNR2E1 patient. **d** Prediction of cells carrying ecNR2E1. **e** Correlation between the expression of *CD24* and *NR2E1* in RNA-seq data from TCGA-GBM patients (n = 166). **f** Pathways involving *NR2E1* among those enriched for upregulated genes in the ecNR2E1 patient. **g** Correlation between the expression of the transcription factors *NR2E1*, *PAX6*, *NFIX*, *SOX5*, and the downstream target *FOXO3*, involved in the regulatory network activated by ecNR2E1, in microarray data from TCGA-GBM patients (n = 526). **h** The transcription factors *NFIX*, *DNMT1*, *SOX5*, and the oncogene *IGF2R*, regulated by *NR2E1*, were upregulated in the ecNR2E1 patient. Statistical significance was assessed using a two-sided Wilcoxon test. **i** In scRNA-seq data of the ecNR2E1 patient, cells expressing *PTPRC* (*CD45*) were annotated as immune cells. **j** In scRNA-seq data of the ecNR2E1 patient, immune cells expressed *SIGLEC10*, a receptor gene for *CD24*.

Supplementary Figure 9.

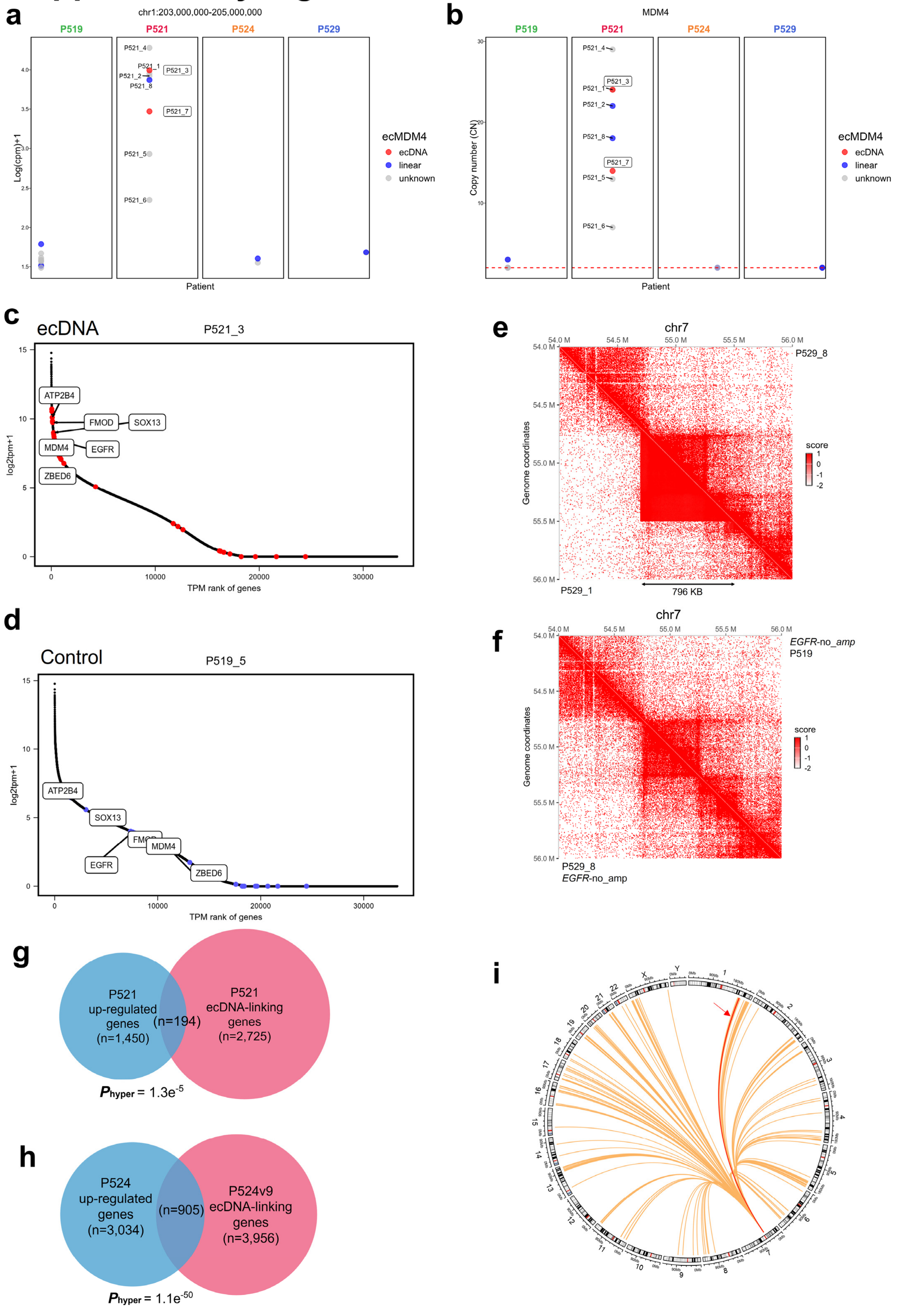

Supplementary Fig. S9.

Heterogeneity characteristics and interactions of ecDNA regions based on Hi-C, WES, and RNA-seq analysis.

a Normalized bulk ATAC-seq signals from ecMDM4 regions across distinct spatial regions in four patients. **b** The copy number of *MDM4* inferred from WES data across distinct spatial regions in four patients. **c** Expression of ecDNA genes within the P521_3 transcriptome. Red dots indicated genes located on ecDNA detected in the P521 patient. **d** Transcriptome in the P519_5, lacking ecDNA. **e, f** Hi-C maps show ecEGFR heterogeneity across different regions in P529, with P529_1 carrying ecEGFR and P529_8 lacking it. **g, h** Hypergeometric test assessing whether genes interacting with the ecEGFR region of P521 and P524_9 were enriched in differentially expressed genes between P521 (ecEGFR) / P524_9 (ecEGFR) and P519 (non-ecEGFR) with a *P*-value < 0.05 considered significant. **i** Genomic regions showing significant *trans*-interactions with ecEGFR after correction for copy number effects, with ecMDM4 links highlighted in red.

Supplementary Figure 10.

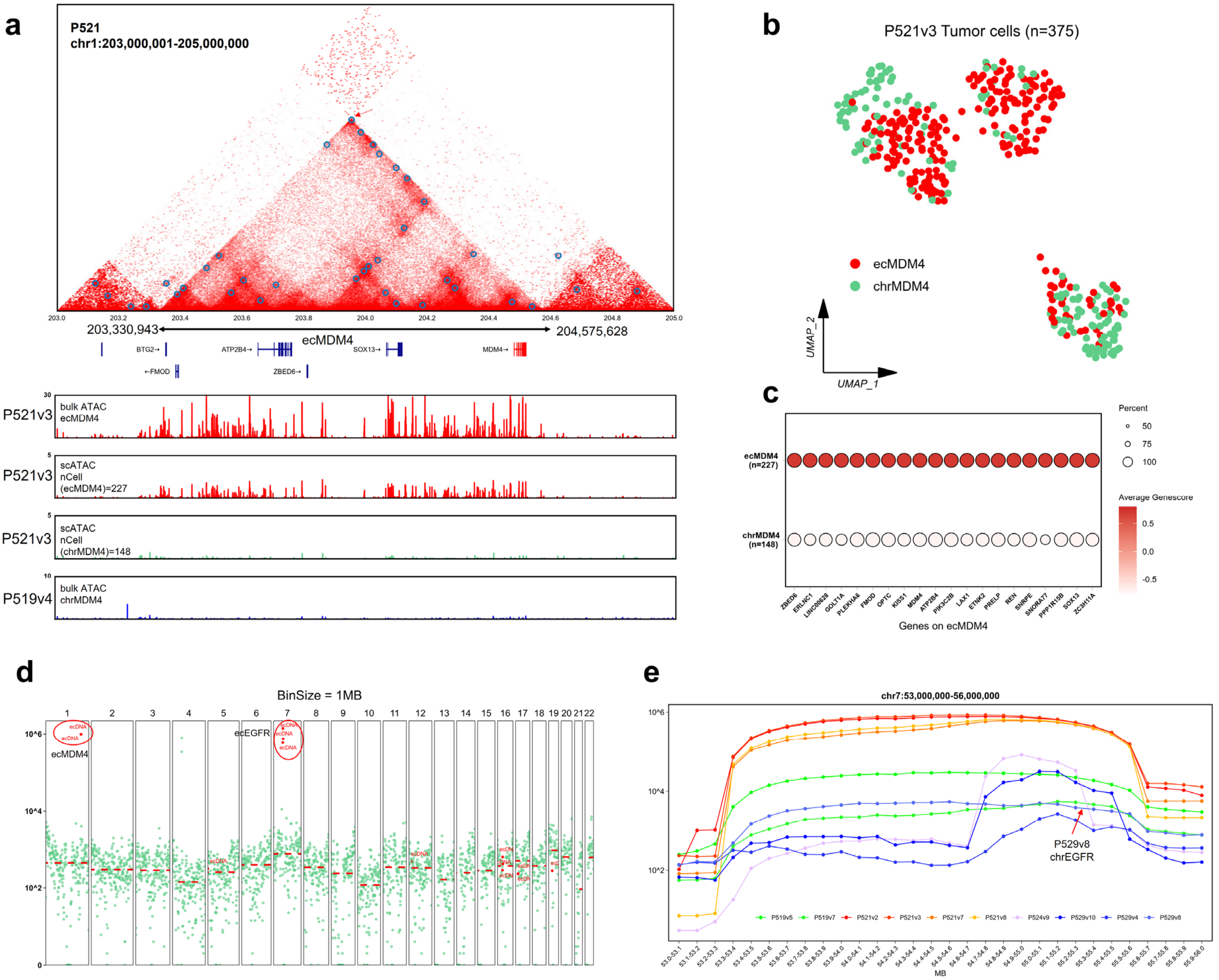

Supplementary Fig. S10.

Interaction frequency within ecDNA regions inferred from scATAC-seq data.

a Hi-C maps showing the regulations within the P521 ecMDM4 region, with circles representing predicted loops. From top to bottom: gene annotations, bulk ATAC-seq signal from P521_3, normalized scATAC-seq signal from ecMDM4-positive cells of P521_3, normalized scATAC-seq signal from non-ecMDM4 cells of P521_3, and bulk ATAC-seq signal from P519_4 (control) without ecMDM4. **b** Dimensionality reduction of cells from P521_3 based on scATAC-seq data, colored by predicted ecMDM4 state. **c** Dotplot showing the GeneScore of ecMDM4-carrying genes in P521_3 cells based on scATAC-seq data, grouped by ecMDM4 state. **d** The genome was divided into 1 Mb bins, and the number of regulations inferred from co-accessibility based on scATAC-seq data was calculated for each bin. Red dots represented bins containing ecDNA, and the red dashed line indicated the average number of regulations per chromosome. **e** The inferred number of regulations within bins for malignant cells based on scATAC-seq data from 10 different regions of four GBM patients, with bins spanning chr7_53,000,000-56,000,000 with a 100 kb sliding window.

Supplementary Figure 11.

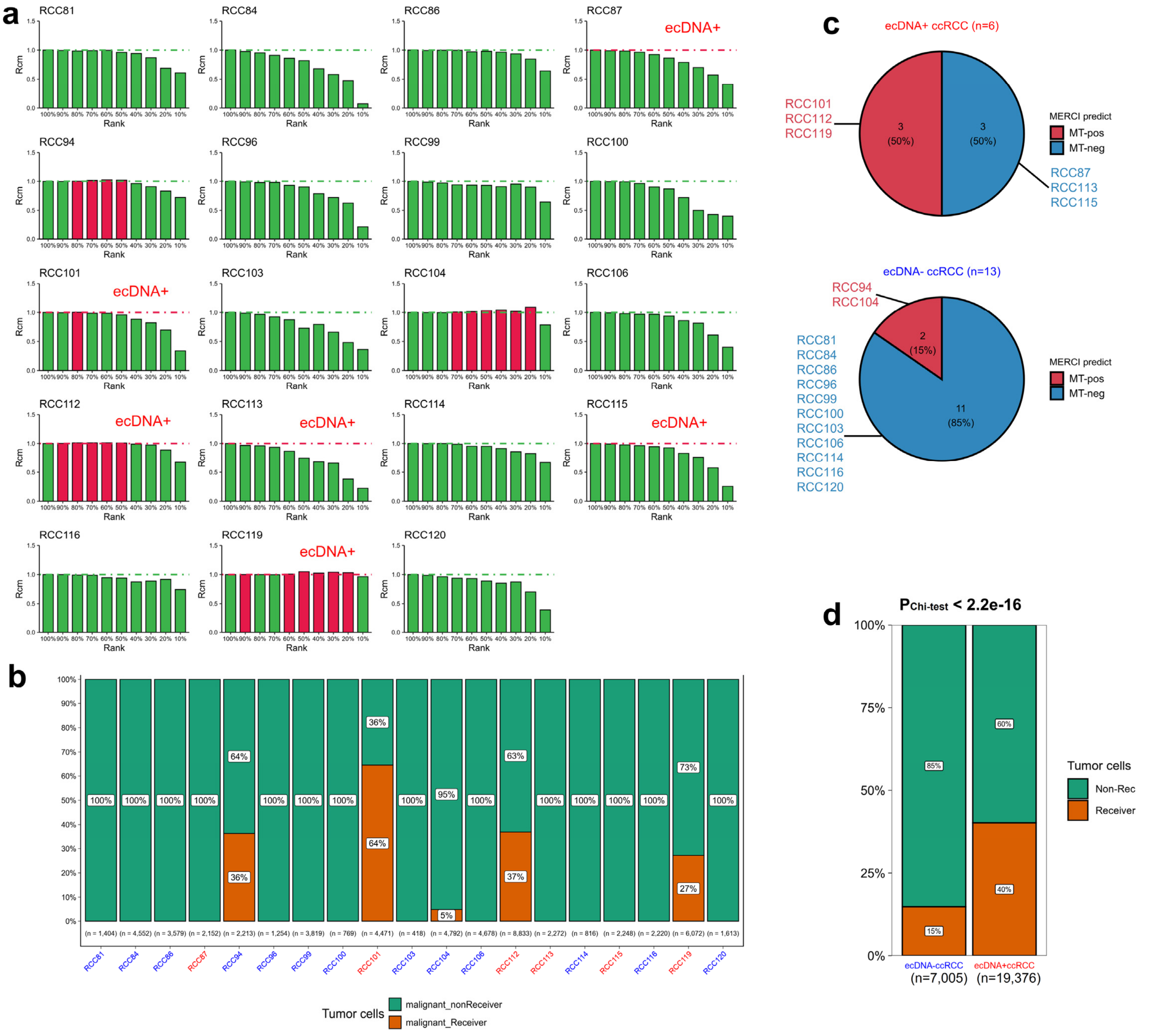

Supplementary Fig. S11.

Prediction of mitochondrial transfer in 19 ccRCC patients and its association with ecDNA classification.

a Significance estimation of the number of positive calls reported by MERCI for 19 ccRCC patients, with ecDNA detected based on scATAC-seq data. **b** Barplot showing the percentage of malignant cells hijacking mitochondria out of all malignant cells across 19 ccRCC patients, with cell types annotated based on scRNA-seq data. Patients marked in red indicated the presence of ecDNA. **c** Pie charts showing the presence of mitochondrial hijacking in ccRCC patients with and without ecDNA. **d** Malignant cells from scRNA-seq data of 19 ccRCC patients were integrated and grouped based on ecDNA detection from scATAC-seq data. The bar graph indicated the percentage of mitochondria-hijacking cells in the ecDNA+ccRCC and ecDNA-ccRCC patients. Chi-squared test was used to assess the association between ecDNA and the mitochondrial transfer phenotype.
